# METTL3-driven m^6^A modification orchestrates mitophagy-dependent ferroptosis in PM2.5-induced lung injury

**DOI:** 10.3389/fimmu.2025.1683819

**Published:** 2025-10-09

**Authors:** Qin Ran, Jie Gao, Guoping Li, Junyi Wang, Xiaolan Li, Anying Xiong, Yi Zhang, Ying Xiong, Xiang He

**Affiliations:** ^1^ Laboratory of Allergy and Precision Medicine, Affiliated Hospital of Southwest Jiaotong University, Chengdu Institute of Respiratory Health, the Third People’s Hospital of Chengdu, Chengdu, China; ^2^ Department of Respiration, Chengdu Third People’s Hospital Branch of National Clinical Research Center for Respiratory Disease, Affiliated Hospital of ChongQing Medical University, Chengdu, China; ^3^ Department of Pulmonary and Critical Care Medicine, Sichuan Friendship Hospital, Chengdu, China; ^4^ National Center for Respiratory Medicine, National Clinical Research Center for Respiratory Disease, State Key Laboratory of Respiratory Disease, Guangzhou Institute of Respiratory Health, The First Affiliated Hospital of Guangzhou Medical University, Guangzhou, China

**Keywords:** PM2.5, METTL3, mitophagy, ferroptosis, lung injury

## Abstract

Air pollution, particularly from fine particulate matter (PM2.5), poses a significant threat to respiratory health, yet the molecular mechanisms underlying PM2.5-induced lung injury remain incompletely understood. This study investigated the role of *N* ^6^-methyladenosine (m^6^A) methyltransferase METTL3 in regulating mitophagy-dependent ferroptosis in bronchial epithelial cells exposed to PM2.5. Using *in vitro* and *in vivo* models, we demonstrated that PM2.5 exposure induced histological alterations in mouse lung tissues, including inflammatory cell infiltration, goblet cell hyperplasia, and mucus hypersecretion, concurrent with enhanced ferroptosis and mitophagy in bronchial epithelial cells. Gain-of-function and loss-of-function experiments showed that METTL3 overexpression exacerbated mitophagy and ferroptosis, while METTL3 silencing attenuated these processes, rescuing cell viability and reducing pulmonary inflammation. *In vivo*, intratracheal administration of METTL3 recombinant protein recapitulated these effects, confirming its role in amplifying PM2.5-induced lung injury. Mechanistically, PM2.5 upregulated METTL3 expression, which promoted *PINK1* mRNA stability through m^6^A modification, activating the PINK1-dependent mitophagy pathway. This led to the excessive clearance of damaged mitochondria, culminating in iron-dependent lipid peroxidation, dysregulation of ferroptosis-related proteins (ACSL4 and xCT), and ferroptotic cell death. Critically, the inhibition of mitophagy with Mdivi-1 protected against histological damage and ferroptosis in mice, underscoring the therapeutic potential of targeting this pathway. Collectively, our findings established a hierarchical regulatory axis where m^6^A–mitophagy–ferroptosis drove lung injury. This study uncovered a novel link between epigenetic modification, mitophagy, and ferroptosis, identifying METTL3-mediated m^6^A modification and mitophagy as potential targets for preventing PM2.5-related respiratory diseases.

## Introduction

The World Health Organization (WHO) reports that nearly 90% of the global population resides in areas with inadequate air quality, with approximately 4.2 million annual premature deaths attributed to ambient outdoor air pollution (1). Among air pollutants, particulate matter ≤2.5 μm (PM2.5) is a key hazard, strongly linked to cardiovascular diseases (2, 3), Alzheimer-related neuropathology (4), and particularly severe impacts on the respiratory system. Epidemiological studies have established associations between PM2.5 exposure and adverse respiratory outcomes, including airway inflammation, declining lung function, and acute exacerbations in chronic obstructive pulmonary disease (COPD) and asthma (5–9). However, the molecular mechanisms underlying PM2.5-induced lung dysfunction remain incompletely understood.

Ferroptosis, a distinct form of regulated cell death, is characterized by iron-dependent phospholipid peroxidation, governed by interconnected metabolic pathways including redox balance, iron homeostasis, mitochondrial function, and amino acid/lipid/glucose metabolism (10). Recent investigations have implicated ferroptosis in PM2.5-mediated lung injury, involving pathways such as Nrf2/SLC7A11/GPX4, PI3K/Akt/Nrf2, and AMPK-Beclin1 (11–13). Concurrently, mitophagy, the selective autophagic clearance of damaged mitochondria, emerges as a critical regulator of cellular homeostasis (14). Preclinical studies have observed mitophagy-dependent ferroptosis in neurodegenerative contexts (15), prompting the exploration of this axis in respiratory pathology, given mitochondria’s vulnerability to PM2.5 and their role in energy metabolism.

The canonical PINK1–PARKIN signaling cascade is central to mitophagy initiation: PINK1 accumulates on dysfunctional mitochondrial outer membranes, recruiting the E3 ubiquitin ligase PARKIN to facilitate organelle degradation (16). Given the pivotal role of PINK1–PARKIN-mediated mitophagy in maintaining mitochondrial health and its potential crosstalk with ferroptosis, defining their roles in PM2.5-induced lung injury is essential for mechanistic clarity. *N* ^6^-Methyladenosine (m^6^A) is the most abundant internal modification in eukaryotic RNAs, and it plays a pivotal role in the biogenesis, processing, and functional regulation of RNA molecules (17). As a core catalytic component of the m^6^A methyltransferase complex, METTL3 has been demonstrated to exert critical functions in diverse biological processes: it mediates m^6^A modification to regulate key post-transcriptional events, including mRNA stability, alternative splicing, and translational efficiency, thereby modulating cellular functions and responses to environmental stressors (18, 19). This established role in stress-responsive RNA regulation positions METTL3 as a promising candidate for mediating pathological responses to PM2.5 exposure. Notably, prior research has linked PM2.5-induced lung damage to the METTL3/YTHDF1 axis. This axis coordinates m^6^A-dependent control of mRNA stability and translational efficiency, which ultimately drives the upregulation of IL-24 expression, a key mediator of PM2.5-associated pulmonary injury (20). Moreover, METTL3 enhanced chemotherapy resistance in small cell lung cancer (SCLC) by activating the Pink1–Parkin mitophagy pathway and exacerbating mitochondrial damage through DCP2 targeting (21). Notably, METTL3 governed PINK1 m^6^A modification via YTHDF2, driving renal tubular epithelial cell apoptosis and mitophagy in Diabetic Kidney Disease (DKD) pathogenesis (22). Here, in this study, we observed increased ferroptosis, mitophagy, and METTL3 expression in both *in vitro* and *in vivo* models of PM2.5 exposure. We therefore aimed to characterize how PM2.5 induces ferroptosis in bronchial epithelial cells, define the role of mitophagy in this process, and elucidate whether METTL3 modulates mitophagy to drive disease-relevant phenotypes. Deciphering these mechanisms not only advances our understanding of PM2.5 pathogenesis but also identifies potential therapeutic targets for preventing or treating PM2.5-related respiratory disorders.

## Methods

### Reagents and antibodies

The following reagents and antibodies were purchased from different companies: ferrostatin-1 (Fer-1; S7243, Selleck, Houston, USA), Mdivi-1 (HY-15886, MCE, New Jersey, USA), Actinomycin-D (HY-17559, MCE, New Jersey, USA), Recombinant Mouse METTL3 (P9262, Fine Test, Wuhan, China), FerroOrange (F374, Dojindo, Kumamoto Prefecture, Japan), Liperfluo (L248, Dojindo, Kumamoto Prefecture, Japan), Mtphagy Dye and Lyso Dye (MD01, Dojindo, Kumamoto Prefecture, Japan), and the EpiQuik™ CUT&RUN m^6^A RNA Enrichment (MeRIP) Kit (P9018, EpiGentek, New York, USA). The primary antibodies used in this study were as follows: rabbit anti-FACL4 antibody (ab155282, Abcam, Cambridge, UK), rabbit anti-METTL3 antibody (ab195352, Abcam, UK), rabbit anti-xCT antibody (DF12509, Affinity, Shanghai, China), rabbit anti-PINK1 antibody (23274-1-AP, Proteintech, Wuhan, China), rabbit anti-PARK2 antibody (14060-1-AP, Proteintech, Wuhan, China), rabbit anti-TOM20 antibody (42406S, CST, Boston, USA), mouse anti-LC3B antibody (83506S, CST, Boston, USA), and mouse anti-β-actin antibody (66009-1-Ig, Proteintech, Wuhan, China), mouse anti- GAPDH (60004-1-Ig, Proteintech, Wuhan, China). The secondary antibodies used in this study were as follows: goat anti-rabbit IgG, HRP-linked antibody (7074, CST, Boston, USA), rabbit anti-mouse IgG H&L (HRP) antibody (ab6728, Abcam, Cambridge, UK), Alexa Fluor 488-conjugated goat anti-Mouse IgG H&L (1:2,000; Abcam, Cambridge, UK), Alexa Fluor 555-conjugated goat anti-Rabbit IgG H&L (1:2,000; Abcam, Cambridge, UK), and Dylight 649 Goat anti-Rabbit IgG antibody (1:1,000; Abbkine, Wuhan, China). PM2.5 sample preparation was described in our previous publication (23). Briefly, PM2.5 was sampled on the rooftop of the 10-story building of Chengdu Third People’s Hospital using an air sampler. Particles were collected on quartz fiber filters for 23 h per working day. Subsequently, the filters were cut into approximately 1-cm^2^ fragments, immersed in sterile water, and ultrasonicated three times to elute water-soluble components. After the lyophilization of the resulting suspension, the PM2.5 extracts were resuspended in phosphate-buffered saline (PBS) and stored at −80°C until subsequent use.

### Animals and experimental protocol

This study was approved by the Animal Ethics Committee of Southwest Jiaotong University. Female C57BL/6 mice, approximately 6–8 weeks old, were purchased from Chongqing Tengxin Biotechnology Co., Ltd. (Chongqing, China) and maintained on a 12-h light/12-h dark cycle at 25°C for 1 week. Then, the mice were randomly divided into the control group (PBS, n = 6) and the PM2.5 exposure group (PM2.5, n = 6). For the PBS group, mice received an equal volume of PBS; for the PM2.5 exposure group, mice were administered PM2.5 (100 μg/mouse) by intratracheal inhalation for 14 consecutive days. On day 15, all mice were sacrificed, and the lung tissues and bronchoalveolar lavage fluid (BALF) were collected and analyzed.

Based on the above experimental grouping, an Mdivi-1 pretreatment and PM2.5 exposure group (Mdivi-1+PM2.5) was added in the subsequent animal model. For the Mdivi-1+PM2.5 group, mice were injected intraperitoneally with Mdivi-1 (10 mg·kg^−1^·day^−1^) and then exposed to PM2.5 (100 μg/mouse) by intratracheal inhalation.

To study the effects of METTL3 on mice, the mice were administered recombinant mouse METTL3 (0.2 µg/50 µL) via intratracheal inhalation for nine consecutive days.

### Cell culture, treatment, and transfection

Beas-2B cells (RRID: CVCL_0168) were purchased from Shanghai Jikai Gene Chemistry Technology Co., Ltd. (Shanghai, China) and maintained in Dulbecco’s modified Eagle’s medium (DMEM) (Thermo Fisher Scientific, Waltham, Massachusetts, USA) containing 10% fetal bovine serum (FBS) (Gibco, Grand Island, New York, USA) and 1% penicillin/streptomycin (Thermo Fisher Scientific, USA). Cells were incubated at 37°C with 5% CO_2_. At 80% confluence, the cells were treated with PM2.5 (62 μg/cm^2^) for 24 h, with or without pretreatment with Fer-1 (10 μM) or Mdivi-1 (20 μM). The synthesized duplex RNAi oligos targeting human *METTL3* or *PINK1* mRNA sequences were designed and transfected into Beas-2B cells using Lipofectamine 2000 according to the manufacturer’s instructions. The siRNA was synthesized by Tsingke Biotechnology Co., Ltd. (Beijing, China).

#### Lentiviral transfection

The lentiviruses containing the packaged plasmids were purchased from GENE Company (Shanghai, China) and used to infect Beas-2B cells for 48 h in the presence of 10 μg/mL polybrene (MCE, HY-112735); then, the stably transduced cell line was screened with puromycin (Beyotime, ST551, Shanghai, China). Finally, the efficiency of overexpression was evaluated using Western blotting and qPCR.

### Cell viability assay

Beas-2B cells were seeded in 96‐well black plates at a density of 5 * 10^3^ per well. The next day, cells were treated with PM2.5 in different concentration gradients for 24 h, 10 μL MTT solution (5 mg/mL, i.e., 0.5% MTT) was added, and incubation was continued for 4 h. Then, the supernatant was discarded, and 150 μL Dimethyl sulfoxide (DMSO) was added and shaken at low speed for 10 min on a shaker to fully dissolve the crystalline material. The absorbance at 490 nm was measured using a microplate reader.

### Quantitative real-time PCR

Total RNA was extracted using the TRIzol reagent (Vazyme, Jiangsu, China). After detecting the purity and concentration of RNA using ScanDrop 100 (Analytik Jena AG, Germany), the RNA was then reverse-transcribed into cDNA by HiScript II Reverse Transcriptase (Vazyme, Jiangsu, China). Next, real-time quantitative PCR was performed using the 2x Taq SYBR Green qPCR Mix (Vazyme, Jiangsu, China). Each sample was tested in triplicate, and the results were calculated using the 2^−ΔΔCT^ and normalized to *GAPDH* mRNA. The primers were synthesized by Tsingke Biotechnology Co., Ltd. (Beijing, China).

### Data collection

The datasets utilized in this study, specifically the Gene Expression Omnibus (GEO) Series entries GSE155617, GSE138870, GSE93329, and GSE182201, were retrieved from the National Center for Biotechnology Information (NCBI) GEO database (http://www.ncbi.nlm.nih.gov/geo/). GMT files corresponding to mitophagy and ferroptosis gene sets were obtained from the Molecular Signatures Database (MSigDB; http://www.gsea-msigdb.org). Enrichment analysis was conducted using the clusterProfiler R package, while correlation analysis was performed using the “cor” function in the R software. Statistical significance was set at a p-value < 0.05.

### MeRIP-qPCR

Total RNA was extracted from Beas-2B cells, and then the MeRIP Kit was used to detect the m^6^A status in mRNA. First, RNA fragments containing m^6^A from total human RNA were captured using the anti-m^6^A antibody, which was incubated with affinity beads on a rotator at room temperature for 90 min. Meanwhile, non-immune IgG was used as a negative control. For each immunoprecipitation, 20 μg of total RNA was prepared, and 2 μg of total RNA was used as input. Subsequently, enriched RNA was released from RNA Binding Beads with elution buffer and purified with ethanol. The relative m^6^A levels in *PINK1* mRNA were determined by normalizing the m^6^A levels obtained from the m^6^A IP with the corresponding gene expression levels from the input samples.

### RNA stability assays

Actinomycin-D (10 μg/mL) was used to detect the lifetime of *PINK1*. Cell samples were harvested at 0, 2, 4, and 6 h after Actinomycin-D treatment. Then, the total RNA was obtained, and the stability of *PINK1* mRNA was determined using qRT-PCR.

### Western blotting

Total protein was extracted from the cells by homogenizing them in Radio Immunoprecipitation Assay Lysis buffer (RIPA) buffer containing 1 mM Phenylmethanesulfonyl fluoride (PMSF) and 1 mM protease inhibitor cocktail. Then, the lysate was placed on ice for 30 min and centrifuged at 12,000 rpm at 4°C for 15 min. Finally, the supernatant was collected, and the protein concentration was measured using a Bicinchoninic Acid Assay (BCA) protein assay kit (Solarbio, Beijing, China). The protein sample was separated by 12% sodium dodecyl sulfate– polyacrylamide gel electrophoresis (SDS–PAGE) (Vazyme, Jiangsu, China) and transferred to polyvinylidene fluoride membrane (PVDF; Millipore, Bedford, Massachusetts, USA). Following the transfer, the membrane was blocked with 5% non-fat milk in TBST (Tris-buffered saline containing 0.1% Tween-20; pH 7.5) for 2 h at room temperature. Subsequently, the membranes were incubated overnight at 4°C in the following primary antibodies: METTL3 (1:1,000), PINK1 (1:1,000), PARKIN (1:1,000), ACSL4 (1:1,000), xCT (1:500), and β-actin (1:10,000). The next day, the membranes were incubated for 2 h at room temperature with anti-mouse and anti-rabbit antibodies. Finally, the membranes were imaged for peroxidase activity of target proteins using Touch Imager (e-Blot, Shanghai, China), and the immune bands were analyzed using the ImageJ software.

### Histology

After fixation and dehydration, the lung tissues were embedded in paraffin and cut into 5-μm-thick sections. Then, the sections were stained with hematoxylin and eosin and periodic acid–Schiff staining reagent according to the manufacturer’s instructions. The pathological changes around the airway were observed using an optical microscope, and the inflammation was scored from 0 to 5 according to our previous methods, with approximately 10 areas scored in a single sample (24). Furthermore, the expression of glycogen in the airway epithelium was also observed; goblet cell proliferation and mucus secretion were also assessed.

### Immunofluorescence staining

Beas-2B cells cultured on 96-well glass plates were fixed with 4% paraformaldehyde, permeabilized with 0.2% Triton X-100, and blocked with 10% goat serum. Then, they were incubated with anti-PINK1 (1:400), anti-PARKIN (1:400), or anti-TOM20 (1:200) and anti-LC3B (1:300) at 4°C overnight and incubated with fluorescent secondary antibodies to bind primary antibodies at room temperature for 1 h. In a similar way, the paraffin tissue sections were dewaxed with xylene, subjected to antigen retrieval in citrate, and blocked with goat serum. Next, the tissue sections were incubated with anti-PINK1 (1:400), anti-PARKIN (1:400), anti-ACSL4 (1:200), anti-xCT (1:100), or anti-TOM20 (1:200) and anti-LC3B (1:300) at 4°C overnight and also treated with fluorescent secondary antibodies the next day. After staining the nuclei with 4′,6-diamidino-2-phenylindole (DAPI), the cell samples were observed using a laser scanning confocal microscope (Olympus, Tokyo, Japan), and the tissue samples were observed using an automatic multispectral scanning microscopy system (Olympus, Tokyo, Japan).

### Transmission electron microscopy

After treatment with PM2.5, the mitochondrial morphology from Beas-2B cells was examined by transmission electron microscopy (TEM; JEM-1400FLASH, JEOL, Tokyo Akishima Station, Japan). Cells were initially fixed with 4% glutaraldehyde and refixed with 1% osmium tetroxide. Following dehydration by acetone step by step, the samples were embedded in epoxy resin, cut into 50-nm slices, and then stained with uranyl acetate and lead citrate. Finally, the specimens were visualized using TEM.

### FerroOrange and Liperfluo staining

Beas-2B cells were seeded in 96-well black plates and cultured overnight. After treatment with PM2.5, the cells were incubated with FerroOrange working solution (F374, Dojindo) and Liperfluo working solution (L248, Dojindo) at 37°C for 30 min. Then, the nuclei were incubated with the Hoechst working solution at 37°C for 15 min. All steps were carried out according to the manufacturer’s instructions. Finally, cells were imaged using a laser scanning confocal microscope (Olympus, FV12-IXCOV).

### Mitophagy detection

Beas-2B cells were seeded in 96-well black plates and treated with PM2.5. Then, the cells were incubated with Mtphagy Dye and Lyso Dye working solution (MD01, Dojindo) at 37°C for 30 min, and nuclei were incubated with Hoechst working solution at 37°C for 15 min. All steps were carried out according to the manufacturer’s instructions. Images were acquired using a laser scanning confocal microscope (Olympus, FV12-IXCOV) and further analyzed using the ImageJ software.

### Statistical analysis

Statistical analyses were conducted using the GraphPad Prism 8.0 software. Data were shown as the mean ± standard error of the mean (SEM) and were derived from a minimum of three independent biological replicates. The unpaired Student’s t-tests and one-way analysis of variance (ANOVA) were used to analyze the data. A p-value <0.05 was considered statistically significant.

## Results

### PM2.5 exposure changed the histological characteristics in mouse lung tissues

**Figure 1:**
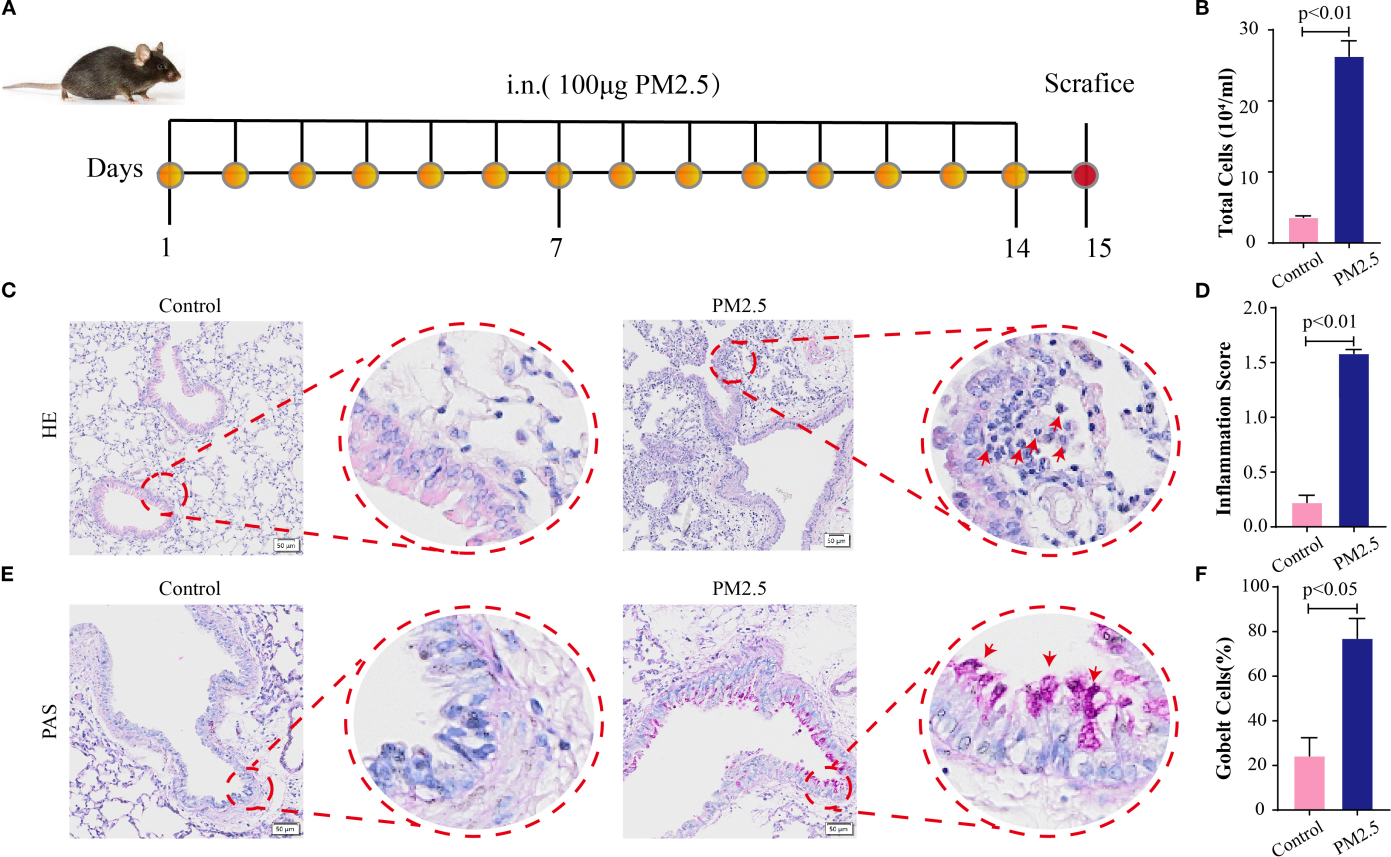
PM2.5 exposure changed the histological characteristics in mouse lung tissues. **(A)** Schematic representation of PM2.5-exposed mouse models’ establishment (n = 6). **(B)** Total cells in BALF in each group. **(C)** H&E stain in lung sections; scale bars = 50 μm. **(D)** The pulmonary inflammation score for each group. **(E)** PAS stain in lung sections; scale bars = 50 μm. **(F)** The percentage of goblet cells in each group. All data presented in this study are representative of at least three independent experiments. Data are presented as the mean ± SEM. PM2.5, particulate matter ≤2.5 μm; BALF, bronchoalveolar lavage fluid; PAS, periodic acid–Schiff.

To assess the histological impact of PM2.5 exposure on murine lung tissues, mouse models were established as outlined in **Figure 1A**. BALF analysis revealed a significant increase in total cell counts in PM2.5-exposed mice compared to the control group (**Figure 1B**). Hematoxylin and eosin (H&E) staining of lung sections demonstrated marked peribronchial inflammatory cell infiltration in the PM2.5 group, with notably denser immune cell accumulation around airways relative to control mice (**Figures 1C, D**). Periodic acid–Schiff (PAS) staining further indicated that PM2.5 exposure induced a significant rise in goblet cell numbers and exacerbated mucus hypersecretion in the airway epithelium, as evidenced by intense PAS-positive staining in the PM2.5 group versus the control (**Figures 1E, F**).

### PM2.5 induced ferroptosis in bronchial epithelial cells

**Figure 2:**
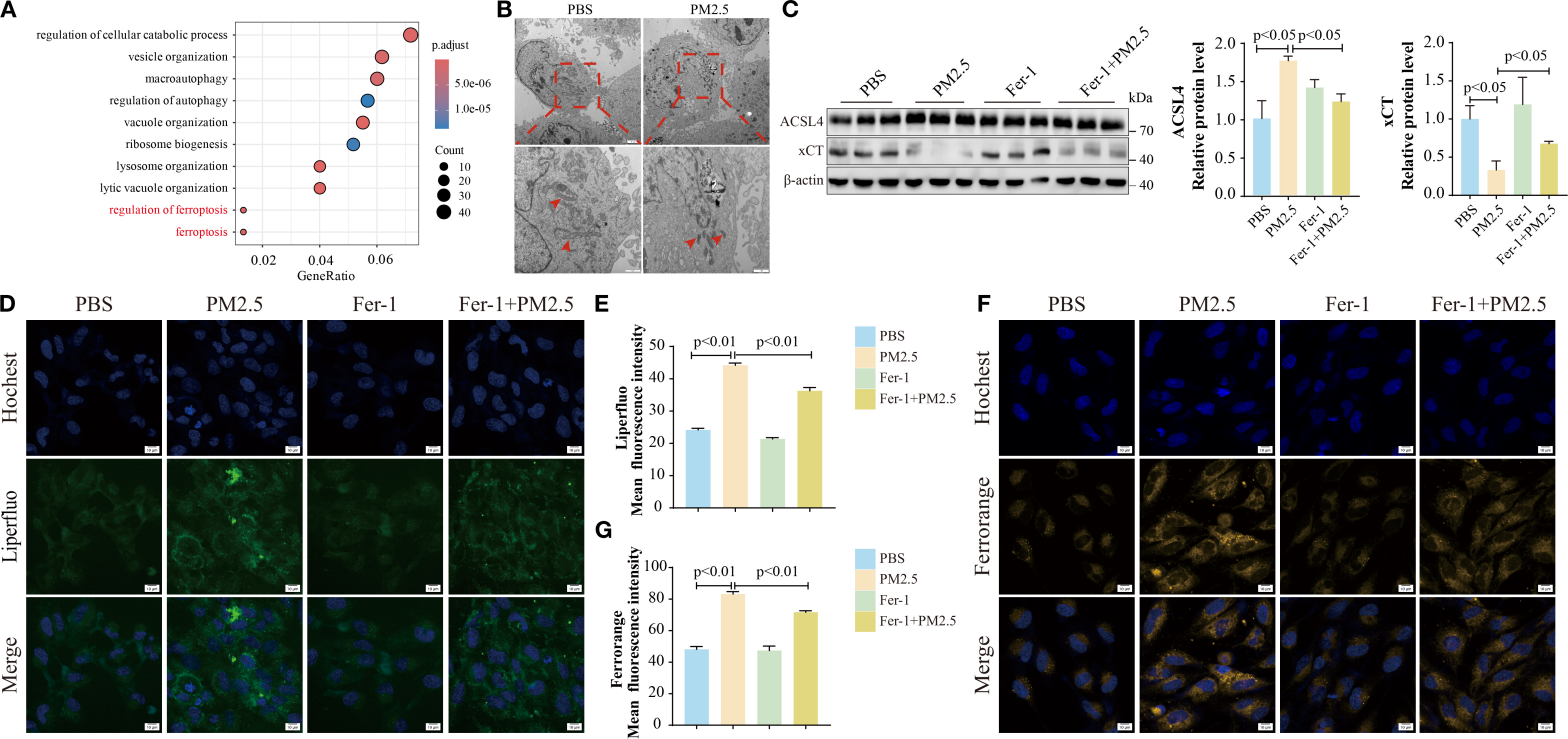
PM2.5 induced ferroptosis in bronchial epithelial cells. **(A)** The bubble plot representing Gene Ontology (GO) analysis of DEGs in Beas-2B cells after PM2.5 exposure. **(B)** Representative TEM images of the morphological changes in mitochondria, and the red arrows indicate the mitochondria; scale bars: top = 2 μm, bottom = 1 μm. **(C)** Western blotting analysis and quantification of ACSL4 and xCT proteins. **(D–G)** Confocal microscopy visualized the alterations in lipid peroxidation and ferrous ions; scale bars = 10 μm. All data presented in this study are representative of at least three independent experiments. Data are presented as the mean ± SEM. PM2.5, particulate matter ≤2.5 μm; DEGs, differentially expressed genes; TEM, transmission electron microscopy.

To further investigate the mechanisms by which PM2.5 induces airway inflammation and mucus secretion, bronchial epithelial cells were exposed to PM2.5 *in vitro*, and RNA-seq analysis was performed. Enrichment analysis of differentially expressed genes revealed a significant association with ferroptosis (**Figure 2A**, **Supplementary Figures S1A–C**). To validate the morphological hallmarks of ferroptosis, TEM was used to examine mitochondrial ultrastructure. TEM analysis showed that PM2.5-treated cells exhibited aberrant mitochondrial morphology, characterized by membrane rupture, reduced volume, and loss or disappearance of cristae, compared to controls (**Figure 2B**). Functional validation demonstrated that the PM2.5-induced decline in cell viability was rescued by Fer-1, a specific ferroptosis inhibitor (**Supplementary Figure S1D**). Key ferroptosis-related proteins ACSL4 and xCT were then evaluated. Both *in vitro* and *in vivo* experiments showed that PM2.5 exposure significantly upregulated ACSL4 protein levels while downregulating xCT (**Figure 2C**, **Supplementary Figure S1E**). Pretreatment with Fer-1 prior to PM2.5 exposure reversed these expression changes, confirming ferroptosis involvement (**Figure 2C**). Given that ferroptosis is associated with intracellular iron accumulation and lipid peroxidation, these parameters were measured using FerroOrange (ferrous iron) and Liperfluo (lipid peroxide) probes in Beas-2B cells. PM2.5 exposure led to significant increases in both ferrous iron and lipid peroxide levels, effects that were potently attenuated by Fer-1 (**Figures 2D–G**). These findings collectively indicate that PM2.5 triggers ferroptosis in bronchial epithelial cells, characterized by mitochondrial structural damage, dysregulated ACSL4/xCT expression, and iron-dependent lipid peroxidation.

### PM2.5 exposure promoted mitophagy-dependent ferroptosis in bronchial epithelial cells

**Figure 3:**
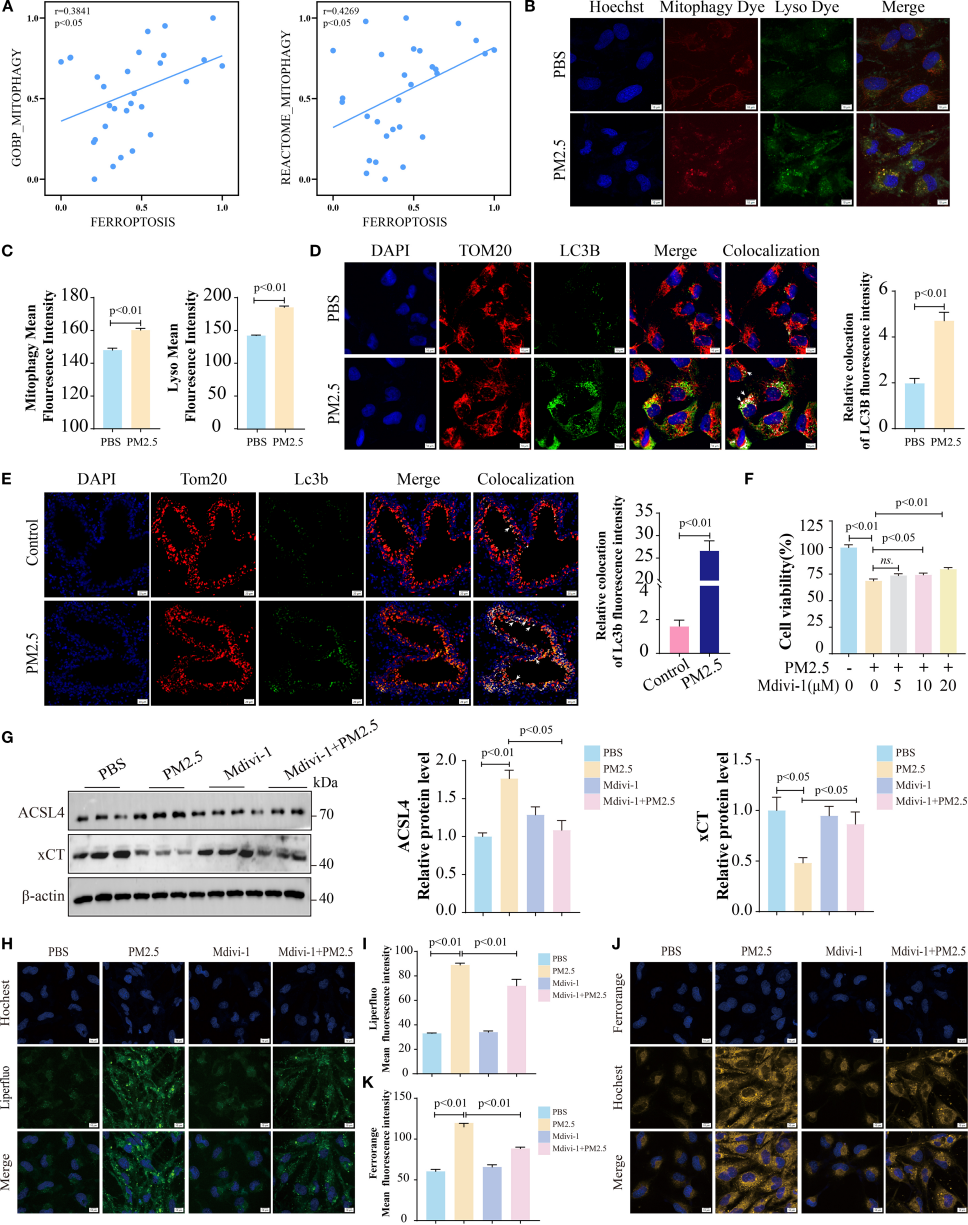
PM2.5 exposure promoted mitophagy-dependent ferroptosis in bronchial epithelial cells. **(A)** Scatter plots showing the correlation between mitophagy and ferroptosis in Beas-2B cells followed by PM2.5 exposure. **(B, C)** Mtphagy Dye and Lyso Dye staining for detection of mitophagy. **(D)** Immunofluorescence staining was performed to examine the expression of TOM20 and LC3B in cell models; arrows, co-localizations of TOM20 with LC3B; scale bars = 10 μm. **(E)** Immunofluorescence staining was performed to examine the expression of Tom20 and Lc3b in mouse models; arrows, co-localizations of Tom20 with Lc3b; scale bars = 20 μm. **(F)** MTT measured the cell viability of Beas-2B cells pretreated with different doses of Mdivi-1 for 2 h, followed by PM2.5 exposure. **(G)** Western blotting analysis of ACSL4 and xCT. **(H, I)** Liperfluo staining for the determination of lipid peroxidation; scale bars = 10 μm. **(J, K)** FerroOrange staining for detection of ferrous ions; scale bars = 10 μm. All data presented in this study are representative of at least three independent experiments. Data are presented as the mean ± SEM. PM2.5, particulate matter ≤2.5 μm.

To build on prior research showing that PM2.5 induces mitophagy-dependent ferroptosis in hippocampal neurons (15), we explored this pathway in bronchial epithelial cells. RNA-seq analysis revealed a significant positive correlation between mitophagy and ferroptosis gene signatures in PM2.5-treated Beas-2B cells (**Figure 3A**). Fluorescence microscopy using Mtphagy Dye and LysoTracker demonstrated enhanced co-localization of mitochondrial and lysosomal signals in PM2.5-exposed cells, indicative of increased mitophagy, compared to PBS controls (**Figures 3B, C**). Immunofluorescent staining for the autophagosome marker LC3B and mitochondrial outer membrane protein TOM20 further confirmed robust co-localization in both Beas-2B cells (**Figure 3D**) and mouse lung tissues (**Figure 3E**) following PM2.5 exposure, reflecting elevated mitophagosome formation. Functionally, the mitophagy inhibitor Mdivi-1 rescued PM2.5-induced reductions in cell viability (**Figure 3F**), concurrently reversing PM2.5-mediated upregulation of the ferroptosis executor ACSL4 and downregulation of xCT (**Figure 3G**). Liperfluo and FerroOrange probes showed that Mdivi-1 pretreatment potently attenuated PM2.5-induced lipid peroxide accumulation and ferrous iron overload (**Figures 3H–K**). Collectively, these data establish that PM2.5 promotes ferroptosis in bronchial epithelial cells through a mitophagy-dependent mechanism.

### METTL3 mediated PM2.5-induced mitophagy-dependent ferroptosis in bronchial epithelial cells

**Figure 4:**
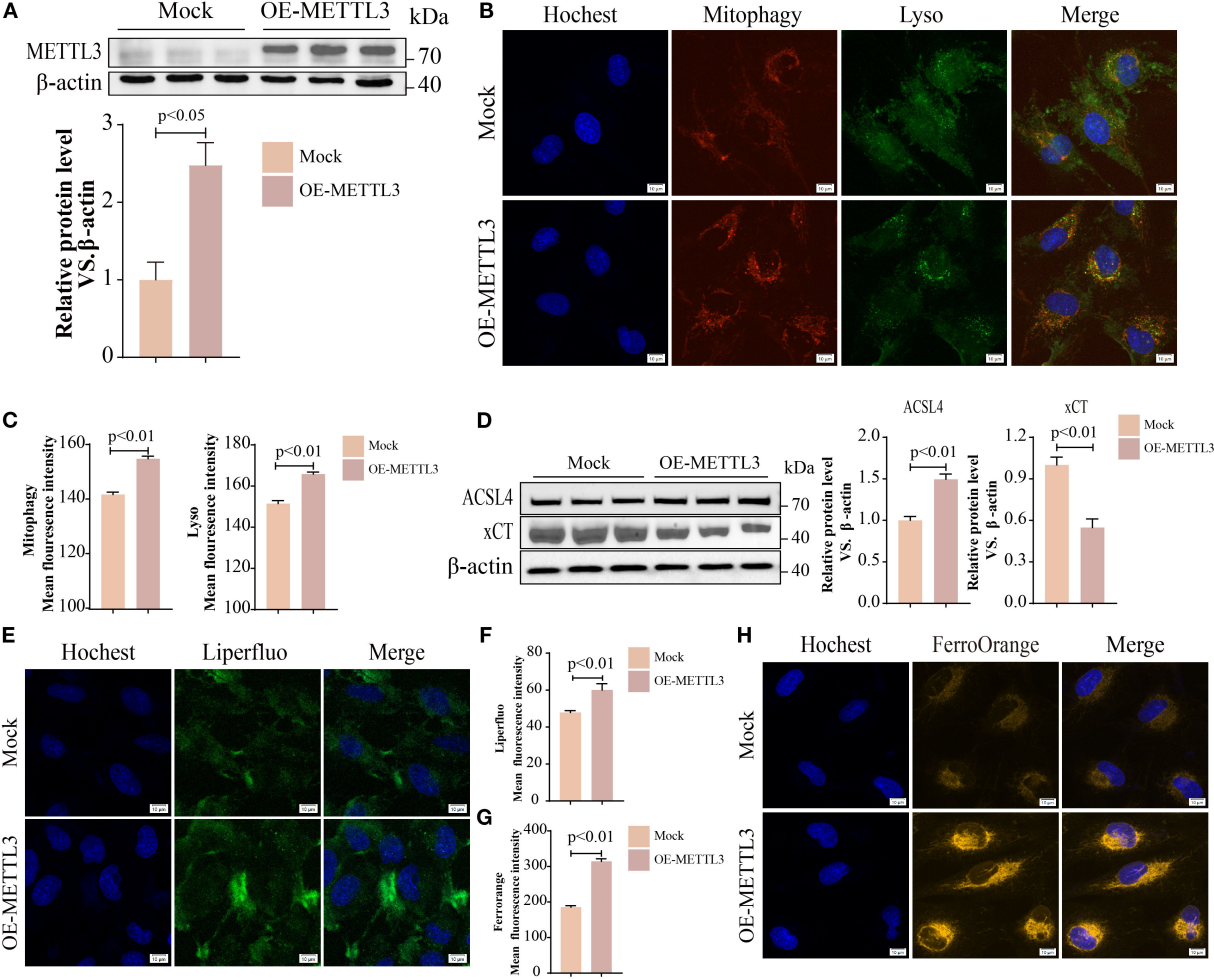
METTL3 mediated PM2.5-induced mitophagy-dependent ferroptosis in bronchial epithelial cells. **(A)** Western blotting analysis of protein levels of METTL3 in OE-METTL3 cells. **(B, C)** Immunofluorescence images and quantification showed Mtphagy and Lyso levels; scale bars = 10 μm. **(D)** Western blotting analysis of ACSL4 and xCT proteins. **(E, F)** Liperfluo determined lipid peroxidation; scale bars = 10 μm. **(G, H)** FerroOrange staining for detection of ferrous ions; scale bars = 10 μm. All data presented in this study are representative of at least three independent experiments. Data are presented as the mean ± SEM. PM2.5, particulate matter ≤2.5 μm.

**Figure 5:**
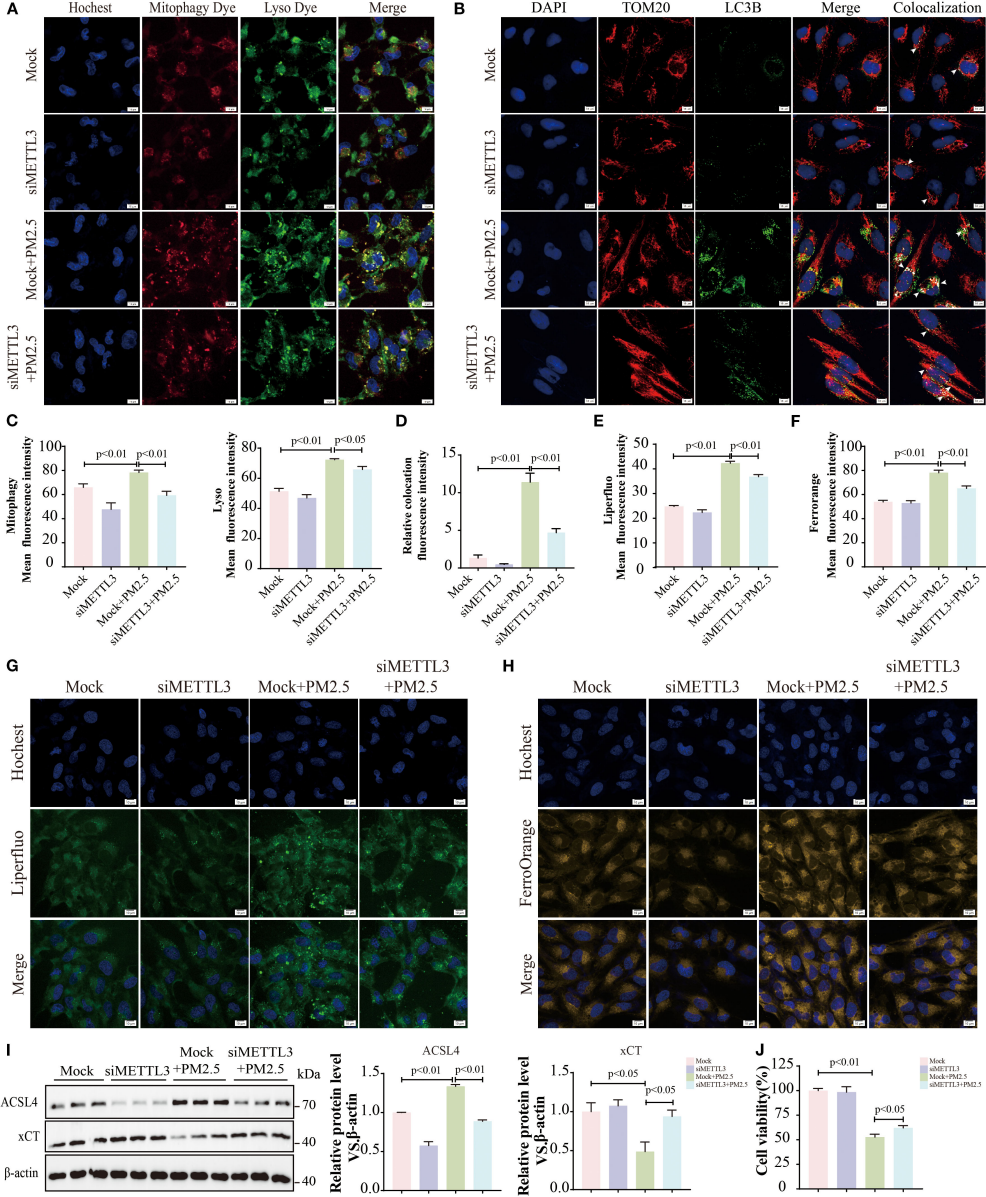
METTL3 mediated PM2.5-induced mitophagy-dependent ferroptosis in bronchial epithelial cells. **(A, C)** Representative immunofluorescence images of Mtphagy Dye and Lyso Dye; scale bars = 10 μm. **(B, D)** Representative immunofluorescent images of TOM20 and LC3B; arrows, co-localizations of TOM20 with LC3B; scale bars = 10 μm. **(E, G)** Representative immunofluorescence images of lipid peroxides; scale bars = 10 μm. **(F, H)** Representative immunofluorescence images of ferrous ions; scale bars = 10 μm. **(I)** Representative immunoblots and quantitative histogram of ACSL4 and xCT. **(J)** The cell viability was measured using MTT. All data presented in this study are representative of at least three independent experiments. Data are presented as the mean ± SEM. PM2.5, particulate matter ≤2.5 μm.

**Figure 6:**
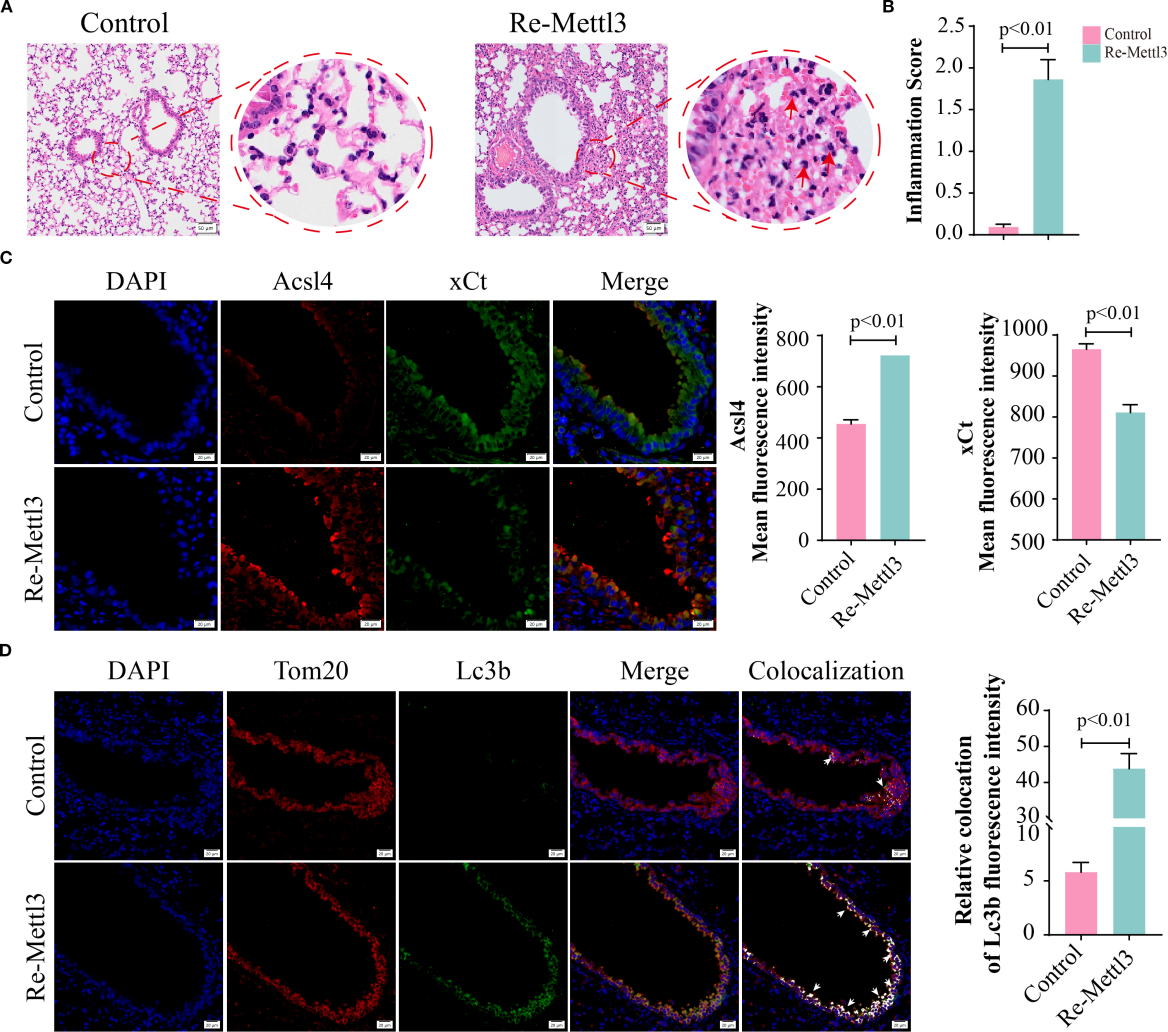
METTL3 mediated PM2.5-induced mitophagy-dependent ferroptosis in bronchial epithelial cells. **(A)** H&E staining in lung sections; scale bars = 50 μm. **(B)** The inflammation scores of pulmonary airway. **(C)** Representative images and quantified mean fluorescence intensity of Acsl4 and xCt in mouse lung tissues from control and Re-Mettl3 groups; scale bars = 20 μm. **(D)** Representative images and quantified mean fluorescence intensity of Tom20 and Lc3b in mouse lung tissues from each group; arrows, co-localizations of Tom20 with Lc3b; scale bars = 20 μm. All data presented in this study are representative of at least three independent experiments. Data are presented as the mean ± SEM. PM2.5, particulate matter ≤2.5 μm.

Evidence has demonstrated that METTL3 plays a pivotal role in the progression of lung injury induced by PM2.5 (20). Then, we validated the expression levels of METTL3 *in vivo* and *in vitro*. Western blotting revealed significant upregulation of METTL3 protein in both mouse lung tissues and Beas-2B cells following PM2.5 exposure (**Supplementary Figures S2A–D**). To characterize the functional role of METTL, we generated a stable Beas-2B cell line overexpressing METTL3 (OE-METTL3) via lentiviral transfection, confirmed by Western blotting and qRT-PCR (**Figure 4A**, **Supplementary Figure S2E**). OE-METTL3 cells showed enhanced mitophagy as indicated by increased co-localization of Mtphagy Dye and LysoTracker signals (**Figures 4B, C**). Meanwhile, these cells exhibited elevated ACSL4 protein levels, reduced xCT expression (**Figure 4D**), and upregulated levels of ACSL4 and SLC7A11 (**Supplementary Figures S2F, G**), accompanied by increased intracellular ferrous iron and lipid peroxide accumulation (**Figures 4E–H**). Subsequently, siRNA-mediated METTL3 knockdown reversed PM2.5-induced mitophagy, as evidenced by diminished Mtphagy/LysoTracker co-localization and reduced LC3B-TOM20 puncta formation (**Figures 5A–D**). METTL3 silencing also attenuated PM2.5-triggered lipid peroxidation and iron overload (**Figures 5E–H**), normalized ACSL4 and xCT expression (**Figure 5I**), and rescued PM2.5-impaired cell viability (**Figure 5J**). *In vivo* validation using intratracheal administration of Mettl3 recombinant protein (Re-Mettl3) in mice showed enhanced airway inflammation (**Figures 6A, B**), upregulated Acsl4, downregulated xCt (**Figure 6C**), and increased Lc3–Tom20 co-localization in lung tissues (**Figure 6D**), consistent with *in vitro* findings. Collectively, these data establish METTL3 as a critical mediator of PM2.5-induced mitophagy-dependent ferroptosis in bronchial epithelial cells.

### METTL3 drove PM2.5-induced mitophagy-dependent ferroptosis through m^6^A-dependent stabilization of *PINK1* mRNA

**Figure 7:**
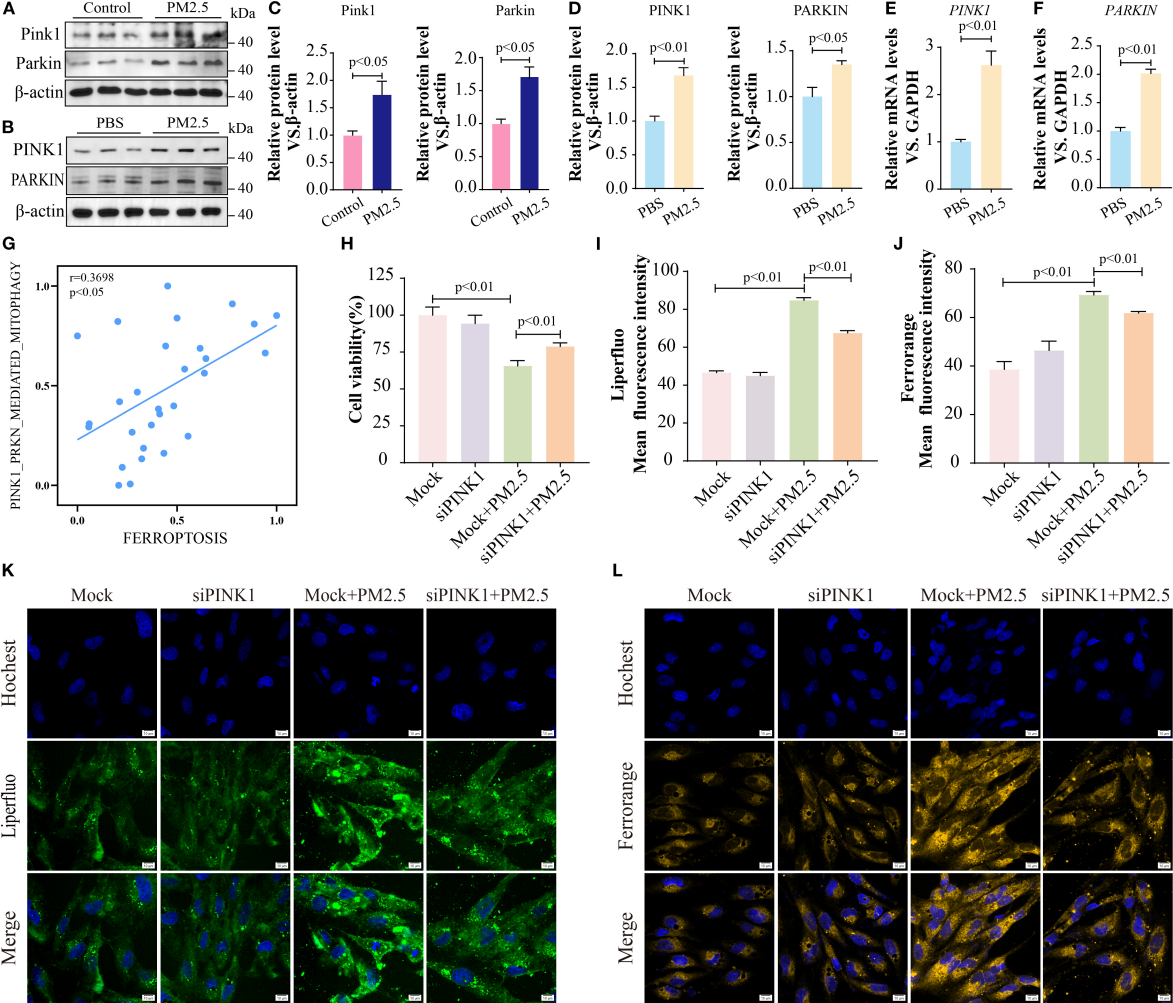
METTL3 drove PM2.5-induced mitophagy-dependent ferroptosis through m^6^A-dependent stabilization of *PINK1* mRNA. **(A, C)** Western blotting determined the protein levels of Pink1 and Parkin in mouse lung tissues. **(B, D)** Western blotting determined the protein levels of PINK1 and PARKIN in Beas-2B cells. **(E, F)** qRT-PCR analysis of *PINK1* and *PARKIN* mRNA levels in Beas-2B cells. **(G)** Scatter plots showing the correlation between mitophagy pathway and ferroptosis following PM2.5 exposure. **(H)** MTT tested the cell viability after inhibiting PINK1. **(I, K)** Representative images of lipid peroxidation after staining LiperFluo Probe in Beas-2B cells following silencing of PINK1; scale bars = 10 μm. **(J, L)** Representative images of ferrous ions after staining FerroOrange Probe in Beas-2B cells following silencing of PINK1; scale bars = 10 μm. All data presented in this study are representative of at least three independent experiments. Data are presented as the mean ± SEM. PM2.5, particulate matter ≤2.5 μm; m^6^A, *N* ^6^-methyladenosine.

Given that PINK1 and PARKIN are critical for initiating mitophagy by mediating mitochondrial degradation (25, 26), we quantified PINK1 and PARKIN expression. Consistent with our hypothesis, PM2.5 exposure significantly upregulated both protein and mRNA levels of PINK1 and PARKIN in both murine lung tissues and Beas-2B cells (**Figures 7A–F**). Meanwhile, PM2.5-induced mitophagy was suppressed after the inhibition of PINK1 (**Supplementary Figure S3**). Furthermore, RNA-seq analysis further revealed a positive correlation between PINK1-mediated mitophagy gene signatures and ferroptosis pathways (**Figure 7G**). Functional validation showed that siRNA-mediated PINK1 knockdown rescued PM2.5-induced reductions in cell viability (**Figure 7H**) and attenuated PM2.5-triggered increases in intracellular ferrous iron and lipid peroxide levels (**Figures 7I–L**), indicating a causal role for PINK1 in linking mitophagy to ferroptosis.

**Figure 8:**
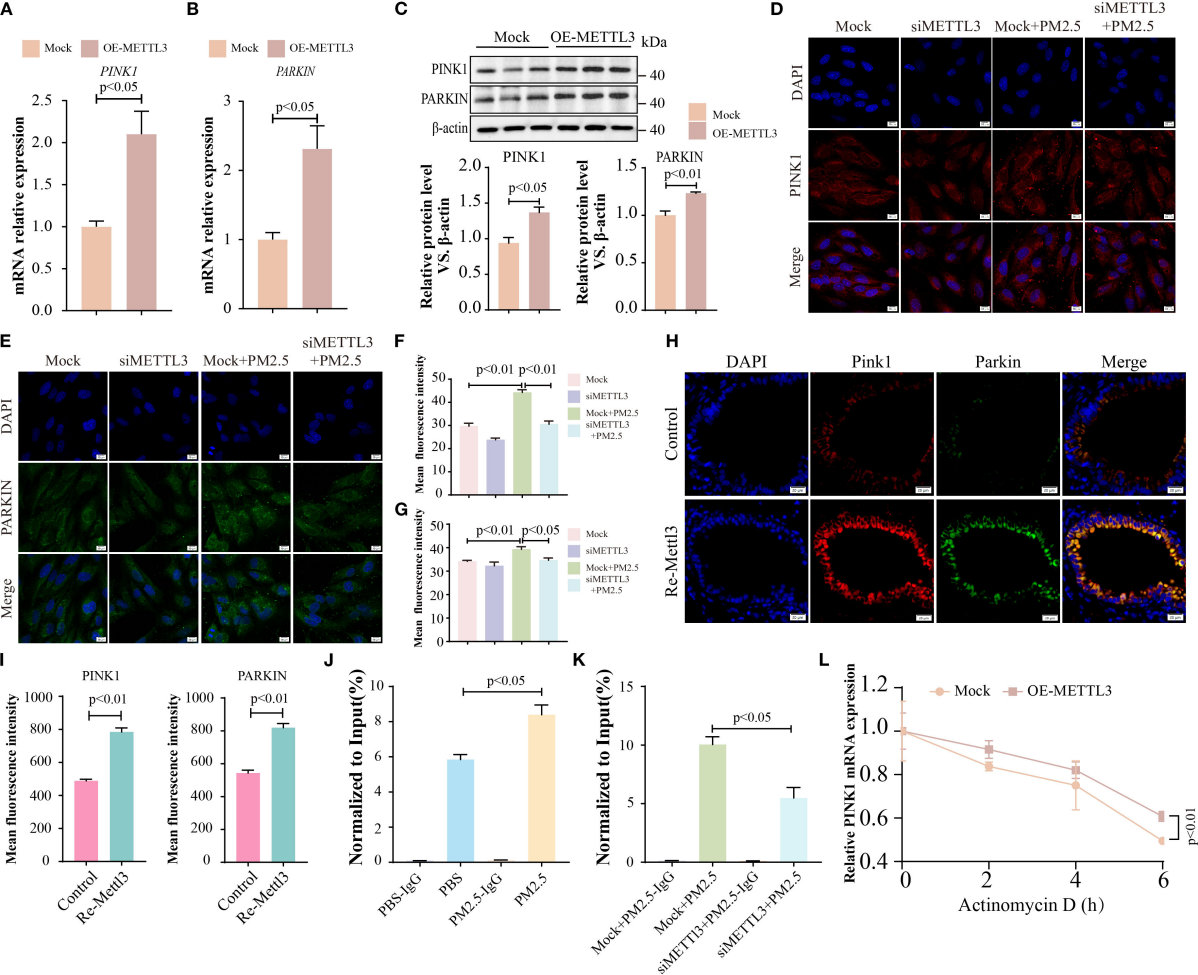
METTL3 drove PM2.5-induced mitophagy-dependent ferroptosis through m^6^A-dependent stabilization of *PINK1* mRNA. **(A, B)** qRT-PCR analysis of the mRNA levels of *PINK1* and *PARKIN* in Beas-2B cells with METTL3 overexpression. **(C)** Western blotting analysis of PINK1 and PARKIN expression in Beas-2B cells with METTL3 overexpression. **(D, E)** Immunofluorescence analysis of PINK1 and PARKIN levels in Beas-2B cells with METTL3 silence; scale bars = 10 μm. **(F, G)** Quantitative analysis of the mean fluorescence intensity of PINK1 and PARKIN. **(H)** Immunofluorescence analysis of PINK1 and PARKIN in lungs in each group; scale bars = 20 μm. **(I)** Quantitative analysis of the mean fluorescence intensity of PINK1 and PARKIN in mice. **(J, K)** The m^6^A enrichment level of *PINK1* was quantified by MeRIP-qPCR among the different experimental groups. **(L)** *PINK1* mRNA levels were analyzed by RT-qPCR assay in OE-METTL3 cells after Actinomycin-D treatment for 0, 2, 4, and 6 **(H)** All data presented in this study are representative of at least three independent experiments. Data are presented as the mean ± SEM. PM2.5, particulate matter ≤2.5 μm.

Next, we explored the regulatory relationship between METTL3 and the two proteins (PINK1 and PARKIN). In METTL3-overexpressing cells, the expression levels of PINK1 and PARKIN were significantly elevated (**Figures 8A–C**), whereas METTL3 knockdown reversed PM2.5-induced upregulation of these proteins (**Figures 8D–G**). *In vivo*, intratracheal administration of Mettl3 recombinant protein increased Pink1 and Parkin expression in mouse lung tissues (**Figures 8H, I**). Given that METTL3 functions as an m^6^A methyltransferase (27), we hypothesized that it modulated *PINK1* and *PARKIN* mRNA stability via m^6^A modification. MeRIP-qPCR analysis confirmed that PM2.5 exposure significantly increased m^6^A enrichment on *PINK1* mRNA (but not *PARKIN* mRNA) in Beas-2B cells, an effect abrogated by METTL3 knockdown (**Figures 8J, K**). Actinomycin-D assays further demonstrated that METTL3 overexpression prolonged *PINK1* mRNA half-life, indicating that METTL3 stabilizes *PINK1* transcripts through m^6^A methylation (**Figure 8L**).

### Mitophagy inhibition alleviates PM2.5-induced histological change in mice

**Figure 9:**
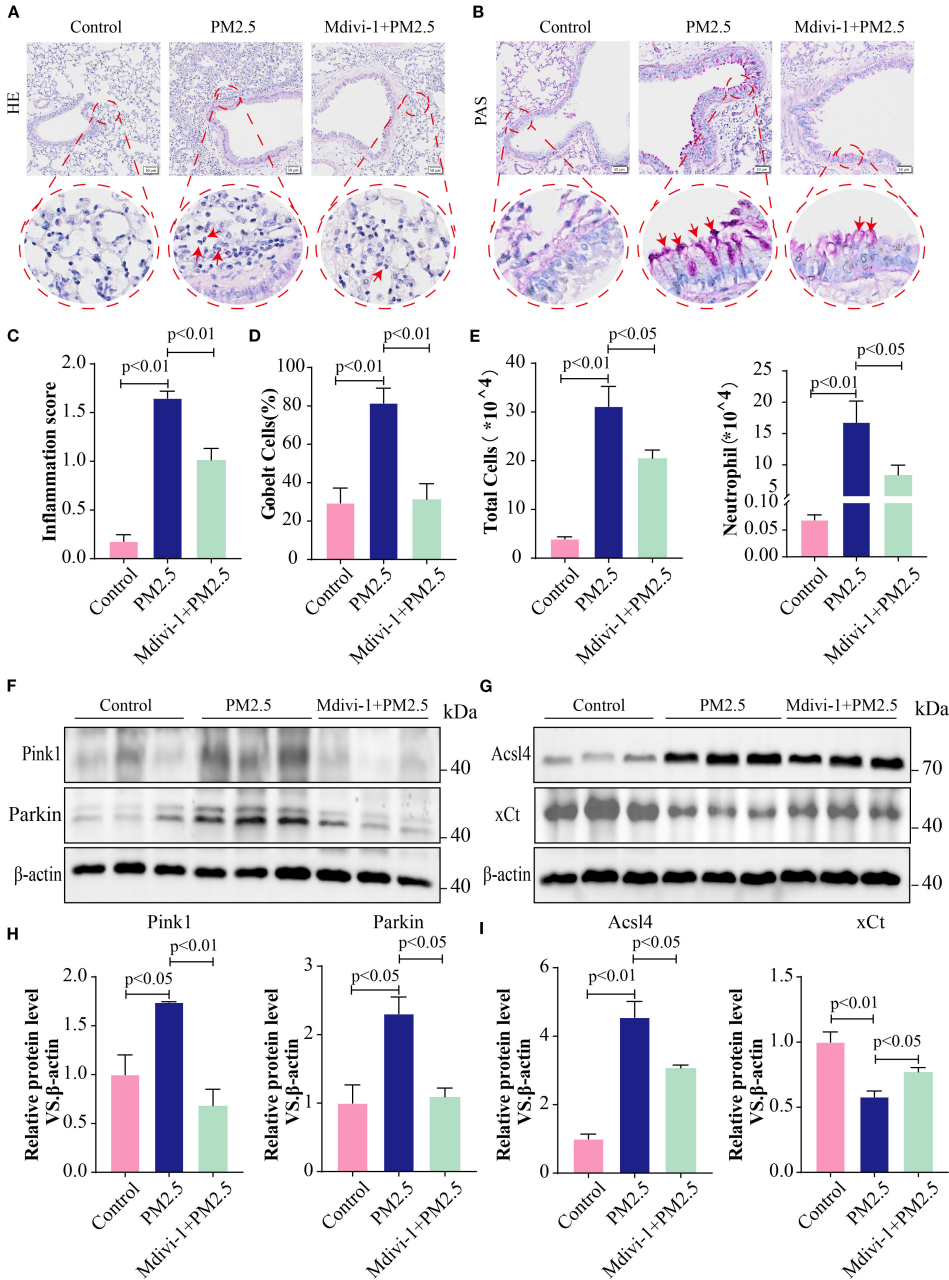
Mitophagy inhibition alleviated PM2.5-induced histological change in mice. **(A)** Lung tissues from different experimental groups were stained with hematoxylin and eosin (H&E); scale bars = 50 μm. **(B)** Representative images of periodic acid–Schiff (PAS) staining; scale bars = 50 μm. **(C)** The pulmonary inflammation score in each group. **(D)** The percentage of goblet cells in each group. **(E)** Total cells and neutrophils in BALF in different experimental groups. **(F–I)** The protein levels of Pink1, Parkin, Acsl4, and xCt in lung tissues in each group. All data presented in this study are representative of at least three independent experiments. Data are presented as the mean ± SEM. PM2.5, particulate matter ≤2.5 μm; BALF, bronchoalveolar lavage fluid.

To further elucidate the impact of mitophagy on mice following PM2.5 exposure, mice were intraperitoneally injected with the mitophagy inhibitor, Mdivi-1. H&E staining demonstrated that, compared to the PM2.5 group, mitophagy inhibition alleviated the degree of pulmonary inflammation and lowered the inflammation score (**Figures 9A, C**). At the same time, PAS staining indicated a decrease in goblet cell numbers and mucus production in the Mdivi-1+PM2.5 group relative to the PM2.5 group (**Figures 9B, D**). Moreover, the inhibition of mitophagy significantly reduced the total cell count and the number of neutrophils in the BALF (**Figure 9E**). Additionally, Western blotting analysis showed that Mdivi-1 downregulated the protein expression levels of Parkin, Pink1, and Acsl4 while upregulating the expression of xCT (**Figures 9F–I**). Collectively, these results suggested that reducing the level of mitophagy suppressed ferroptosis in lung tissues and protected bronchial epithelial cells from damage in mice exposed to PM2.5.

## Discussion

**Figure 10:**
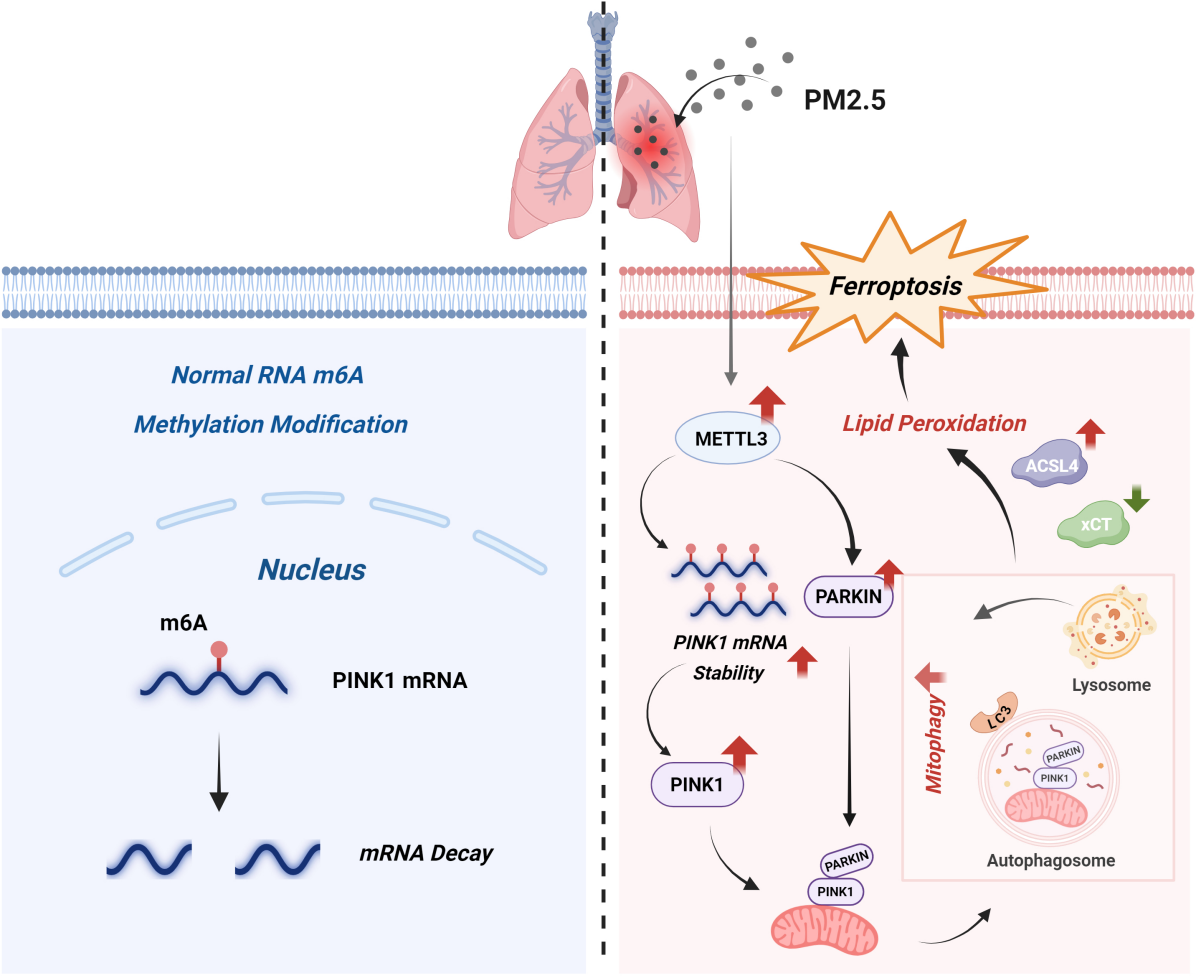
Schematic model. Under homeostatic conditions (left), the normal m^6^A modification of PINK1 mRNA promotes its degradation, thereby sustaining basal mitophagy levels. Upon PM2.5 exposure (right), on the one hand, upregulated METTL3 expression enhances m^6^A modification of PINK1 mRNA; this modification stabilizes the transcript and consequently increases PINK1 protein levels. On the other hand, METTL3 also increases PARKIN expression. PINK1 then recruits PARKIN, subsequently triggering mitophagy. Excessive mitophagy disrupts redox homeostasis, inducing lipid peroxidation, which is characterized by elevated ACSL4 expression and reduced xCT expression, and ultimately drives ferroptosis, a central pathogenic event in PM2.5-induced lung injury. m^6^A, *N* ^6^-methyladenosine; PM2.5, particulate matter ≤2.5 μm.

PM2.5 exposure is strongly linked to the pathogenesis of diverse diseases, including pulmonary, cardiovascular, and neurological disorders. Previous studies have implicated PM2.5-induced lung injury in m^6^A modification dysregulation (20), ferroptosis activation (28), and mitochondrial dynamic alterations (29). However, the mechanistic interplay between m^6^A methylation, mitophagy, and ferroptosis in PM2.5-mediated lung injury remains poorly defined. Our study established that METTL3, an m^6^A methyltransferase, orchestrated mitophagy-dependent ferroptosis in bronchial epithelial cells by stabilizing *PINK1* mRNA through m^6^A modification. Upon PM2.5 challenge, bronchial epithelial cells rapidly upregulate METTL3 expression, which promotes *PINK1* mRNA stability and subsequent activation of mitophagy, leading to the intracellular accumulation of ferrous iron and lipid peroxides. These convergent events ultimately culminated in ferroptotic cell death in bronchial epithelial cells (**Figure 10**). Our study employed a well-characterized PM2.5 preparation to ensure reproducibility; however, we recognize that real-world PM2.5 exhibits considerably different composition depending on geography, season, and emission sources. Variability in metal content, organic fractions, or endotoxin levels could influence the mechanistic effect and nature of the observed biological pathway (30). Therefore, we view our findings as an important baseline and suggest that future work should compare PM2.5 samples with different physicochemical profiles to better assess the generalizability of these results.

Previous investigations have linked PM2.5-induced lung damage to ferroptosis via multiple pathways, including the Nrf2/SLC7A11/GPX4 axis and PI3K/Akt-mediated Nrf2 upregulation (12, 31). Consistent with these findings, our study observed heightened airway inflammation and mucus hypersecretion in response to PM2.5 exposure. Importantly, we found that PM2.5 induced ferroptosis in bronchial epithelial cells. This was evidenced by mitochondrial structural damage, dysregulated ACSL4/xCT expression, and iron-dependent lipid peroxidation. In the context of PM2.5 exposure, the induction of ferroptosis may disrupt the normal function of bronchial epithelial cells, compromising the airway barrier and facilitating the entry of pathogens and toxic substances. The increase in lipid peroxidation products and intracellular iron levels not only reflects the occurrence of ferroptosis but also suggests that oxidative stress plays a crucial role in this process.

Growing evidence highlights crosstalk between mitophagy and ferroptosis in disease contexts. For example, doxorubicin-induced cardiotoxicity involves GPx4 downregulation and mitochondria-dependent ferroptosis (32), while TDCPP-mediated neurotoxicity relies on PINK1/PARKIN-driven mitophagy to promote ferroptosis (33). Our prior work also demonstrated that PM2.5 exacerbates hippocampal neuron injury through HO-1-mediated mitophagy-dependent ferroptosis (15). We also demonstrated that PM2.5 exposure promoted mitophagy-dependent ferroptosis in bronchial epithelial cells. RNA-seq analysis revealed a positive correlation between mitophagy and ferroptosis gene signatures. Enhanced mitophagy, as indicated by increased co-localization of mitochondrial and lysosomal signals and elevated mitophagosome formation, was observed in PM2.5-treated cells. The inhibition of mitophagy by Mdivi-1 rescued cell viability, reversed the expression of ferroptosis-related proteins, and attenuated lipid peroxidation and iron overload. This suggests that mitophagy, although initially a protective mechanism for clearing damaged mitochondria, may be dysregulated by PM2.5, ultimately contributing to ferroptosis. In normal physiological conditions, mitophagy helps maintain mitochondrial quality and function, but under PM2.5 stress, excessive or dysregulated mitophagy may lead to the release of iron from damaged mitochondria, fueling lipid peroxidation and promoting ferroptosis. Hence, the inhibition of mitophagy by small-molecule agents shows therapeutic potential against PM2.5-mediated lung injury, warranting further investigation to establish the pharmacological basis for clinical application.

A substantial and growing body of research has established that m^6^A modification plays a crucial role in inflammatory responses through its interactions with diverse m^6^A-associated factors. METTL3, the primary enzyme responsible for m^6^A modification, has been reported to have elevated expression in cases of lung injury (20, 34). Additionally, neutrophil extracellular traps (NETs) have been shown to facilitate the transcription of METTL3-driven mitochondrial reprogramming, which amplifies ferroptosis during secondary acute lung injury (35). Consistent with these prior findings, our study also observed an upregulation of METTL3 levels and ferroptosis levels following PM2.5 exposure *in vivo* and *in vitro*. Recent literature has further demonstrated that histone lactylation-regulated METTL3 promotes ferroptosis and exacerbates sepsis-associated lung injury, while pharmacological blockade of GPR81 signaling emerges as a viable strategy to disrupt lactate-induced METTL3 overexpression, thereby preserving alveolar epithelial integrity through ferroptosis inhibition and mitigating lung injury progression (28). Moreover, METTL3 has been implicated in different acute kidney injury (AKI) models as well as in human biopsies and cultured tubular epithelial cells (TECs), and the METTL3-targeted inhibitor Cpd-564 has demonstrated efficacy in ameliorating renal dysfunction and suppressing inflammation (36). Collectively, these findings demonstrate that METTL3 plays a pivotal role in the pathogenesis of those inflammatory diseases and represents a promising therapeutic target for their treatment. Based on our previous experimental results and literature reports, we hypothesized that PM2.5 may induce lung injury by upregulating METTL3, which then modulates the levels of mitophagy-dependent ferroptosis in bronchial epithelial cells. To test this hypothesis, we conducted gain-of-function and loss-of-function experiments by overexpressing and silencing METTL3 in cells exposed to PM2.5. As anticipated, overexpression of METTL3 led to an increase in mitophagy levels. Concurrently, ferroptosis levels were also elevated, as indicated by decreased cell viability, changes in the protein and mRNA levels of ACSL4 and xCT, and increased accumulation of lipid peroxides and ferrous ions. Moreover, administration of the METTL3 recombinant protein in mice led to a significant increase in lung inflammation and alterations in the levels of ferroptosis-related proteins in lung tissues. Conversely, silencing METTL3 resulted in a reduction in both mitophagy and ferroptosis levels. Therefore, our results demonstrate that METTL3 plays a critical role in PM2.5-induced mitophagy-dependent ferroptosis of bronchial epithelial cells. Targeting METTL3 or its downstream regulatory pathways may offer a novel therapeutic approach for the clinical management of environmental pollution-associated lung injury. Consequently, elucidating the downstream regulatory mechanisms of METTL3 constitutes a critical unmet need in current research.

PINK1 and PARKIN form a canonical signaling axis that drives mitophagy, the selective autophagic degradation of damaged mitochondria (37). Our study demonstrated upregulated expression of both proteins after PM2.5 exposure and METTL3 overexpression. Meanwhile, METTL3 silencing blunted PM2.5-induced PINK1 and PARKIN upregulation. METTL3, as an m^6^A methyltransferase, is known to regulate mRNA stability, splicing, and translation (21, 22). MeRIP-qPCR analysis confirmed that PM2.5 exposure increased m^6^A enrichment on *PINK1* transcripts, an effect abrogated by METTL3 knockdown. Moreover, our results showed that METTL3 modulated *PINK1* mRNA stability through m^6^A modification. PM2.5 exposure increased m^6^A enrichment on *PINK1* mRNA through METTL3, and METTL3 overexpression prolonged its half-life. This finding provides a novel molecular mechanism by which METTL3 regulates mitophagy-dependent ferroptosis. The m^6^A-dependent stabilization of *PINK1* mRNA by METTL3 may lead to increased PINK1 protein levels, promoting mitophagy initiation.

## Conclusion

In summary, our study establishes a hierarchical regulatory model where m^6^A–mitophagy–ferroptosis represents a key axis driving lung injury. These findings not only deepen our understanding of the complex interplay between air pollution, epigenetic modification, and regulated cell death but also identify the METTL3-mediated m^6^A modification and mitophagy-dependent ferroptosis as promising targets for preventing or treating PM2.5-related respiratory diseases.

## Data Availability

The original contributions presented in the study are included in the article/**Supplementary Material**. Further inquiries can be directed to the corresponding author.

## Supplementary material

Supplementary Figure 1
**(A-C)** The bubble plot representing KEGG enrichment analysis (A), Reactome pathway over-representation analysis **(B)** and WikiPathways over-representation analysis **(C)** of DEGs in Beas-2B cells after PM2.5 exposure; **(D)** MTT measured the cell viability of Beas-2B cells were pretreated with different doses of Fer-1 for 2 h followed by PM2.5 exposure; (H) Western blot analysis of ACSL4 and xCT; All data presented in this study are representative of at least three independent experiments. Data are presented as the mean ± SEM.

Supplementary Figure 2
**(A, C)** Western blot analysis of Mettl3 in mice lung tissue; **(B, D)** Western blot analysis of METTL3 in cells (B and D); (E and F) Western blot analysis to verify the silencing efficiency of METTL3 in Beas-2B cells; **(G)** qRT-PCR analysis to verify the overexpression efficiency of *METTL3* in Beas-2B cells; **(H)** The mRNA levels of *ACSL4* and *SLC7A11* in OE-METTL3 cells. All data presented in this study are representative of at least three independent experiments. Data are presented as the mean ± SEM.

Supplementary Figure 3
**(A, C)** Immunofluorescence analysis of PINK1 in each group; **(B, D)** Representative images and quantified mean fluorescence intensity of TOM20 and LC3B in each group. All data presented in this study are representative of at least three independent experiments. Data are presented as the mean ± SEM.

## References

[1] Losacco C Perillo A. Particulate matter air pollution and respiratory impact on humans and animals. Environ Sci pollut Res Int. (2018) 25:33901–10. doi: 10.1007/s11356-018-3344-9, PMID: 30284710

[2] Zaręba Ł Piszczatowska K Dżaman K Soroczynska K Motamedi P Szczepański MJ. The relationship between fine particle matter (PM2.5) exposure and upper respiratory tract diseases. J Pers Med. (2024) 14:98. doi: 10.3390/jpm14010098, PMID: 38248800 PMC10817350

[3] Hayes RB Lim C Zhang Y Cromar K Shao Y Reynolds HR. PM2.5 air pollution and cause-specific cardiovascular disease mortality. Int J Epidemiol. (2020) 49:25–35. doi: 10.1093/ije/dyz114, PMID: 31289812 PMC7124502

[4] Thiankhaw K Chattipakorn N Chattipakorn SC. PM2.5 exposure in association with AD-related neuropathology and cognitive outcomes. Environ pollut. (2022) 292:118320. doi: 10.1016/j.envpol.2021.118320, PMID: 34634399

[5] Davel AP Lemos M Pastro LM Pedro SC de André PA Hebeda C. Endothelial dysfunction in the pulmonary artery induced by concentrated fine particulate matter exposure is associated with local but not systemic inflammation. Toxicology. (2012) 295:39–46. doi: 10.1016/j.tox.2012.02.004, PMID: 22361244

[6] Habre R Moshier E Castro W Nath A Grunin A Rohr A. The effects of PM2.5 and its components from indoor and outdoor sources on cough and wheeze symptoms in asthmatic children. J Expo Sci Environ Epidemiol. (2014) 24:380–7. doi: 10.1038/jes.2014.21, PMID: 24714073

[7] Nachman KE Parker JD. Exposures to fine particulate air pollution and respiratory outcomes in adults using two national datasets: a cross-sectional study. Environ Health. (2012) 11:25. doi: 10.1186/1476-069X-11-25, PMID: 22490087 PMC3361500

[8] Montoya-Estrada A Torres-Ramos YD Flores-Pliego A Ramirez-Venegas A Ceballos-Reyes GM Guzman-Grenfell AM. Urban PM2.5 activates GAPDH and induces RBC damage in COPD patients. Front Biosci (Schol Ed). (2013) 5:638–49. doi: 10.2741/S396, PMID: 23277075

[9] Gleason JA Bielory L Fagliano JA. Associations between ozone, PM2.5, and four pollen types on emergency department pediatric asthma events during the warm season in New Jersey: a case-crossover study. Environ Res. (2014) 132:421–9. doi: 10.1016/j.envres.2014.03.035, PMID: 24858282

[10] Jiang X Stockwell BR Conrad M. Ferroptosis: mechanisms, biology and role in disease. Nat Rev Mol Cell Biol. (2021) 22:266–82. doi: 10.1038/s41580-020-00324-8, PMID: 33495651 PMC8142022

[11] Berent H. Blood platelet adrenaline and noradrenaline levels in patients with pheochromocytoma. Pol Tyg Lek. (1987) 42:515–8. doi: 10.1016/j.intimp.2022.109186, PMID: 3615280

[12] Wang Y Shen Z Zhao S Huang D Wang X Wu Y. Sipeimine ameliorates PM2.5-induced lung injury by inhibiting ferroptosis via the PI3K/Akt/Nrf2 pathway: A network pharmacology approach. Ecotoxicol Environ Saf. (2022) 239:113615. doi: 10.1016/j.ecoenv.2022.113615, PMID: 35567927

[13] Pavia C Cabezas P Albarran JM. What do teachers know about diabetes mellitus? Med Clin (Barc). (1987) 89:5–6. doi: 10.1016/j.ecoenv.2022.114083, PMID: 3613741

[14] Liu J Wang J Xiong A Zhang L Zhang Y Liu Y. Mitochondrial quality control in lung diseases: current research and future directions. Front Physiol. (2023) 14:1236651. doi: 10.3389/fphys.2023.1236651, PMID: 37538379 PMC10395103

[15] Li X Ran Q He X Peng D Xiong A Jiang M. HO-1 upregulation promotes mitophagy-dependent ferroptosis in PM2.5-exposed hippocampal neurons. Ecotoxicol Environ Saf. (2024) 277:116314. doi: 10.1016/j.ecoenv.2024.116314, PMID: 38642409

[16] Su L Zhang J Gomez H Kellum JA Peng Z. Mitochondria ROS and mitophagy in acute kidney injury. Autophagy. (2023) 19:401–14. doi: 10.1080/15548627.2022.2084862, PMID: 35678504 PMC9851232

[17] Louloupi A Ntini E Conrad T Ørom UAV. Transient N-6-methyladenosine transcriptome sequencing reveals a regulatory role of m6A in splicing efficiency. Cell Rep. (2018) 23:3429–37. doi: 10.1016/j.celrep.2018.05.077, PMID: 29924987

[18] Xiang Y Laurent B Hsu CH Nachtergaele S Lu Z Sheng W. RNA mA methylation regulates the ultraviolet-induced DNA damage response. Nature. (2017) 543:573–6. doi: 10.1038/nature21671, PMID: 28297716 PMC5490984

[19] Yao L Li G Xiong A Liu J Zeng R Zhang L. Fine particulate matter exacerbates asthma by activating STC2-mediated mitophagy through METTL3/YTHDF2-dependent m6A methylation. J Hazard Mater. (2025) 495:138854. doi: 10.1016/j.jhazmat.2025.138854, PMID: 40499413

[20] He X Zhang L Liu S Wang J Liu Y Xiong A. Methyltransferase-like 3 leads to lung injury by up-regulation of interleukin 24 through N6-methyladenosine-dependent mRNA stability and translation efficiency in mice exposed to fine particulate matter 2.5. Environ pollut. (2022) 308:119607. doi: 10.1016/j.envpol.2022.119607, PMID: 35718042

[21] Sun Y Shen W Hu S Lyu Q Wang Q Wei T. METTL3 promotes chemoresistance in small cell lung cancer by inducing mitophagy. J Exp Clin Cancer Res. (2023) 42:65. doi: 10.1186/s13046-023-02638-9, PMID: 36932427 PMC10022264

[22] Wang F Bai J Zhang X Wang D Zhang X Xue J. METTL3/YTHDF2 m6A axis mediates the progression of diabetic nephropathy through epigenetically suppressing PINK1 and mitophagy. J Diabetes Investig. (2024) 15:288–99. doi: 10.1111/jdi.14113, PMID: 38013600 PMC10906015

[23] He X Zhang L Hu L Liu S Xiong A Wang J. PM2.5 aggravated OVA-induced epithelial tight junction disruption through fas associated via death domain-dependent apoptosis in asthmatic mice. J Asthma Allergy. (2021) 14:1411–23. doi: 10.2147/JAA.S335590, PMID: 34848976 PMC8612670

[24] Zhang L He X Xiong Y Ran Q Xiong A Wang J. Transcriptome-wide profiling discover: PM2.5 aggravates airway dysfunction through epithelial barrier damage regulated by Stanniocalcin 2 in an OVA-induced model. Ecotoxicol Environ Saf. (2021) 220:112408. doi: 10.1016/j.ecoenv.2021.112408, PMID: 34111662

[25] Quinn PMJ Moreira PI Ambrósio AF Alves CH. PINK1/PARKIN signalling in neurodegeneration and neuroinflammation. Acta Neuropathol Commun. (2020) 8:189. doi: 10.1186/s40478-020-01062-w, PMID: 33168089 PMC7654589

[26] Ramirez A Old W Selwood DL Liu X. Cannabidiol activates PINK1-Parkin-dependent mitophagy and mitochondrial-derived vesicles. Eur J Cell Biol. (2022) 101:151185. doi: 10.1016/j.ejcb.2021.151185, PMID: 34915361 PMC8816654

[27] Jiang X Liu B Nie Z Duan L Xiong Q Jin Z. The role of m6A modification in the biological functions and diseases. Signal Transduct Target Ther. (2021) 6:74. doi: 10.1038/s41392-020-00450-x, PMID: 33611339 PMC7897327

[28] Wu D Spencer CB Ortoga L Zhang H Miao C. Histone lactylation-regulated METTL3 promotes ferroptosis via m6A-modification on ACSL4 in sepsis-associated lung injury. Redox Biol. (2024) 74:103194. doi: 10.1016/j.redox.2024.103194, PMID: 38852200 PMC11219935

[29] Liu Q Weng J Li C Feng Y Xie M Wang X. Attenuation of PM(2.5)-induced alveolar epithelial cells and lung injury through regulation of mitochondrial fission and fusion. Part Fibre Toxicol. (2023) 20:28. doi: 10.1186/s12989-023-00534-w, PMID: 37464447 PMC10353144

[30] Jia YY Wang Q Liu T. Toxicity research of PM(2.5) compositions *in vitro* . Int J Environ Res Public Health. (2017) 14:232. doi: 10.3390/ijerph14030232, PMID: 28245639 PMC5369068

[31] Wang X Wang Y Huang D Shi S Pei C Wu Y. Astragaloside IV regulates the ferroptosis signaling pathway via the Nrf2/SLC7A11/GPX4 axis to inhibit PM2.5-mediated lung injury in mice. Int Immunopharmacol. (2022) 112:109186. doi: 10.1016/j.intimp.2022.109186, PMID: 36115280

[32] Tadokoro T Ikeda M Ide T Deguchi H Ikeda S Okabe K. Mitochondria-dependent ferroptosis plays a pivotal role in doxorubicin cardiotoxicity. JCI Insight. (2020) 5. doi: 10.1172/jci.insight.132747, PMID: 32376803 PMC7253028

[33] Qian B Jiang RJ Song JL Wang CQ. Organophosphorus flame retardant TDCPP induces neurotoxicity via mitophagy-related ferroptosis *in vivo* and *in vitro* . Chemosphere. (2022) 308:136345. doi: 10.1016/j.chemosphere.2022.136345, PMID: 36087716

[34] Jia J Yuan Y He Y Wasti B Duan W Chen Z. Inhibition of METTL3 alleviated LPS-induced alveolar epithelial cell apoptosis and acute lung injury via restoring neprilysin expression. Life Sci. (2023) 333:122148. doi: 10.1016/j.lfs.2023.122148, PMID: 37805166

[35] Zhang H Wu D Wang Y Guo K Spencer CB Ortoga L. METTL3-mediated N6-methyladenosine exacerbates ferroptosis via m6A-IGF2BP2-dependent mitochondrial metabolic reprogramming in sepsis-induced acute lung injury. Clin Transl Med. (2023) 13:e1389. doi: 10.1002/ctm2.1389, PMID: 37715457 PMC10504453

[36] Wang JN Wang F Ke J Li Z Xu CH Yang Q. Inhibition of METTL3 attenuates renal injury and inflammation by alleviating TAB3 m6A modifications via IGF2BP2-dependent mechanisms. Sci Transl Med. (2022) 14:eabk2709. doi: 10.1126/scitranslmed.abk2709, PMID: 35417191

[37] Imberechts D Kinnart I Wauters F Terbeek J Manders L Wierda K. DJ-1 is an essential downstream mediator in PINK1/parkin-dependent mitophagy. Brain. (2022) 145:4368–84. doi: 10.1093/brain/awac313, PMID: 36039535 PMC9762950

