# Supplementary figures and images for: METTL3-driven m6A modification orchestrates mitophagy-dependent ferroptosis in PM2.5-induced lung injury
Source: Front Immunol. 2025 Oct 9;16:1683819. doi: 10.3389/fimmu.2025.1683819 (PMC12545146; doi:10.3389/fimmu.2025.1683819)

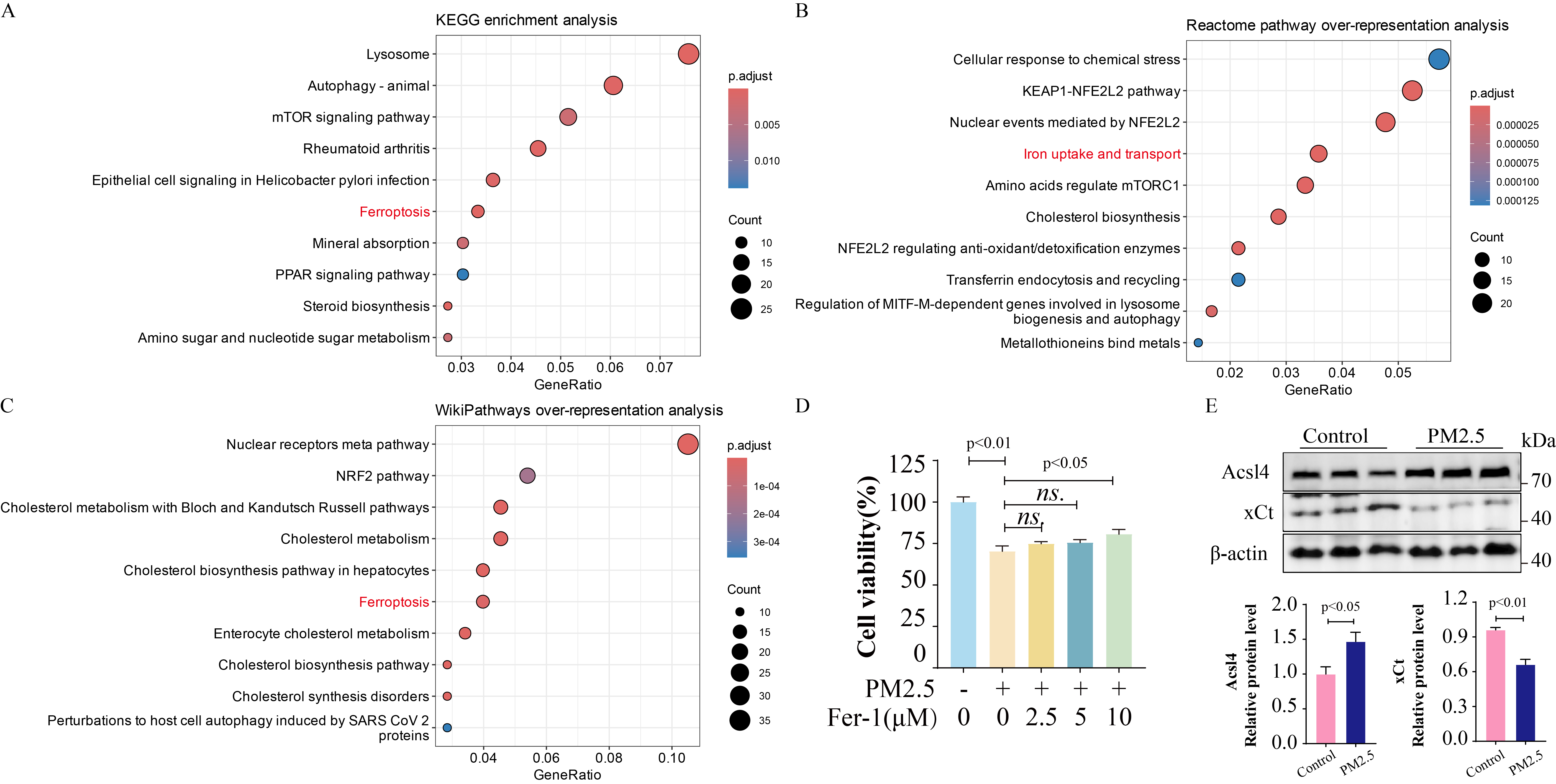

Supplement: Supplementary Figure 1 — (A-C) The bubble plot representing KEGG enrichment analysis (A), Reactome pathway over-representation analysis (B) and WikiPathways over-representation analysis (C) of DEGs in Beas-2B cells after PM2.5 exposure; (D) MTT measured the cell viability of Beas-2B cells were pretreated with different doses of Fer-1 for 2 h followed by PM2.5 exposure; (H) Western blot analysis of ACSL4 and xCT; All data presented in this study are representative of at least three independent experiments. Data are presented as the mean ± SEM. [file Image1.jpeg]

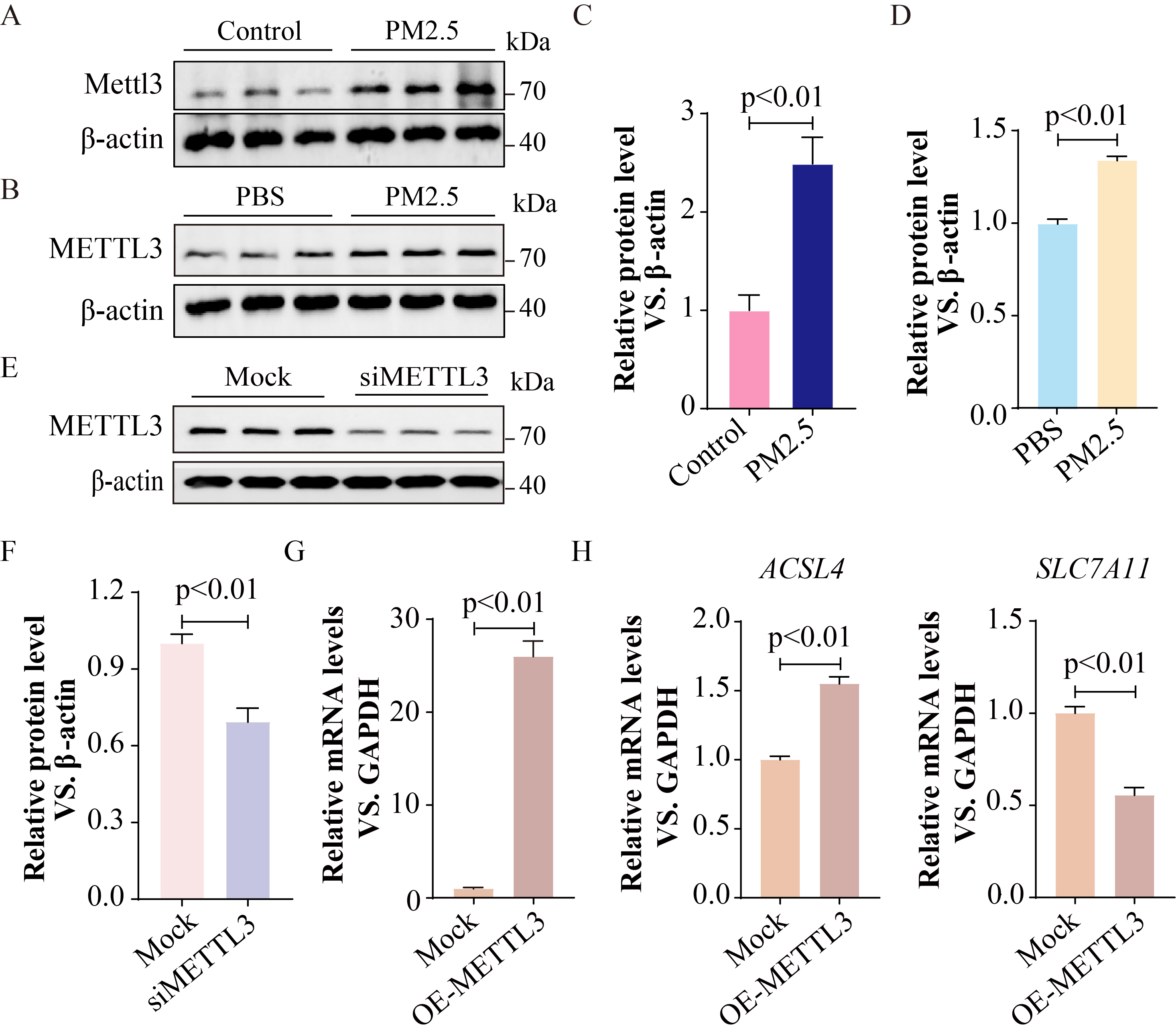

Supplement: Supplementary Figure 2 — (A, C) Western blot analysis of Mettl3 in mice lung tissue; (B, D) Western blot analysis of METTL3 in cells (B and D); (E and F) Western blot analysis to verify the silencing efficiency of METTL3 in Beas-2B cells; (G) qRT-PCR analysis to verify the overexpression efficiency of METTL3 in Beas-2B cells; (H) The mRNA levels of ACSL4 and SLC7A11 in OE-METTL3 cells. All data presented in this study are representative of at least three independent experiments. Data are presented as the mean ± SEM. [file Image2.jpeg]

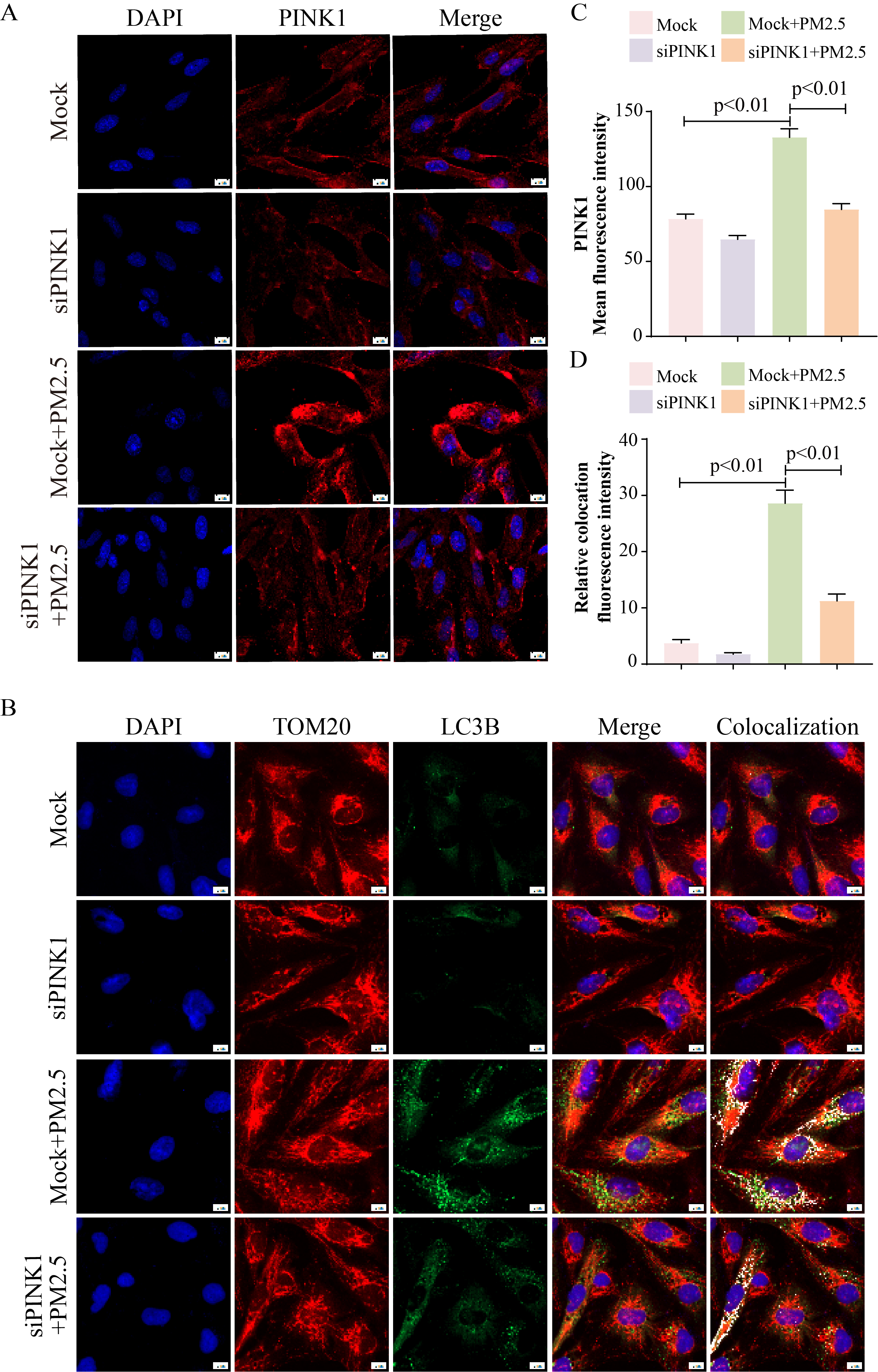

Supplement: Supplementary Figure 3 — (A, C) Immunofluorescence analysis of PINK1 in each group; (B, D) Representative images and quantified mean fluorescence intensity of TOM20 and LC3B in each group. All data presented in this study are representative of at least three independent experiments. Data are presented as the mean ± SEM. [file Image3.jpeg]
